# Inheritance of fruit yield and quality in melon (*Cucumis melo* L.) grown under field salinity stress

**DOI:** 10.1038/s41598-019-43616-6

**Published:** 2019-05-10

**Authors:** Mahmoud Akrami, Ahmad Arzani

**Affiliations:** Department of Agronomy and Plant Breeding, College of Agriculture, Isfahan University of Technology, Isfahan, 84156-83111 Iran

**Keywords:** Plant breeding, Salt

## Abstract

Cultivation of salinity-tolerant crops can help mitigate salinization threats to soil and fresh water resources. This study was conducted to investigate the quantitative genetic basis of yield, yield components, and quality-related traits of salinity-stressed melon (*Cucumis melo* L.) using 55 melon hybrids and their 11 parents (half diallel). The results of combined ANOVA revealed highly significant effects of salinity and genotype on all the traits studied. Salinity stress influenced the inheritance of all the traits. The lower values of variance components (mainly additive), GCA/SCA ratio, as well as broad- and narrow-sense heritabilities were estimated for saline conditions (EC_W_ = 14 dSm^−1^) when compared with those obtained under non-saline conditions. Fruit weight was governed by additive effects in non-saline conditions, but largely governed by the dominant nature in saline conditions. Based on the results obtained, especially as reflected by fruit yield, it is possible to develop melon hybrids with higher salinity tolerance than is currently observed in tolerant cultivars. On the other hand, most of the traits contributing to fruit quality are found to be governed by additive effects, allowing for their further improvement through recurrent selection to develop new cultivars of high yield and good quality for cultivation under saline conditions.

## Introduction

Among the major abiotic stresses, salinity stress poses a serious yield-limiting problem to agriculture and crop production the world over, especially in arid and semi-arid regions^1^. Iran, as the third world producer of melon^2^, has about 34 million ha of salt-affected land area which includes 4.1 million ha of irrigated land^3^ leading to an annual economic loss of almost US$ 1 billion^4^.

Endogenous saline soils and salinity stress arising from saline water irrigation^5^ adversely affect melon growth and production. In such circumstances, the ultimate target of breeding programs will be to enhance crop yield performance^1^. Melon, as a moderately, salinity-tolerant crop species, is cultivated in saline field conditions having different yield loss profiles depending on severity of salinity stress, genotype, and environmental conditions^5–9^. In addition to declining fruit yield, salinity stress lessens some fruit morphology attributes such as size^10,11^ and flesh (pulp) percentage^12–14^. On the other hand, fruit sweetness in melon increases because of salinity stress^8,9^.

Salinity stress is one of the most salient environmental factors hindering plant growth and productivity^1,15^. Nevertheless, knowledge of the genetic nature of the desired trait likely to be under selection in a saline environment is a prerequisite to the satisfactory prediction of genotypic responses to salinity stress^1^. In addition, genes contribute to variations in response to environmental stresses, eventually influence performance and productivity of stressedplants, and are critical for adaptation to unfavorable conditions^16^. It follows that it is not only important to exploit natural variations in crops for abiotic stress adaptation, but also equally important to understand how these variations are affected by various gene actions and how efficiently they can be exploited in plant-breeding programs particularly for adaptation to harsh environmental conditions^1^. It is, thus, most profitable to understand the architecture of quantitative traits such as salinity tolerance for developing salinity-tolerant melon cultivars.

The quantitative genetics of yield and yield-related traits in melon under non-saline conditions have been extensively studied^17–21^. Moreover, a number of studies have reported on the combining ability and heterosis effects in fruit yield and other morphological traits of melon under non-saline conditions^17,18,22–25^. Few studies have, however, been devoted tothe inheritance of fruit quality traits in melon. Feyzian *et al*.^17^ studied the inheritance of yield in melon using a diallel design with seven cultivars and found that it was mainly governed by additive gene effects under non-saline conditions. In a similar study, Pouyesh *et al*.^18^ found that both additive and non-additive gene actions contributed to the inheritance of fruit number and yield under non-saline conditions. Total soluble solids (TSS) index has been used as a quality parameter to determine sweetness, ripening, and marketability of melon fruits. While inheritance of TSS in melon grown under non-saline conditions has been already investigated^22,26,27^, the genetic basis of TSS under saline conditions still remains to be explored.

Another perceived shortcoming involves studies of the genetic structure of the yield and quality related traits under salinity stress on which only scant information is available. It is, therefore, the objective of the present study to investigate the genetics of yield, yield components, and quality related traits of progenies derived from a 11 × 11 diallel cross grown under saline field conditions. The findings can have important implications for improving melon cultivars for cultivation in saline soils.

## Results

### Fruit yield and components

The results of combined ANOVA revealed highly significant effects of salinity stress on melon yield and its components (Table 3). In addition, genotypes (parents and hybrids) differed significantly in these traits. While genotype × salinity interaction was significant for fruit weight (FWT) and fruit yield (FY), those of general combining ability (GCA) × salinity and specific combinig ability (SCA) × salinity were significant for FWT and FY. GCA and SCA had highly significant (*P* < 0.01) effects on all the studied traits (Table 3).

The results of GCA and SCA effects, components of genetic variance, GCA/SCA ratio, and heritability estimates of the traits are reported in Table 4 for each of the environmental conditions investigated. GCA and SCA effects on FY and its components were significant under both non-saline and salinity stress conditions, showing that both additive and non-additive gene effects are associated with governing these traits (Table 4). In non-saline conditions, the GCA/SCA ratio was high (0.75) for FWT but low for number of fruit (NF) and FY (0.36 and 0.41, respectively). Similarly, narrow sense heritability (${h}_{n}^{2}$) estimates were high for FWT (0.69) but relatively low for NF (0.25) and FY (0.37) (Table 4). Broad-sense heritability estimates were high for these traits in non-saline conditions (0.92, 0.71, and 0.92 for FWT, NF, and FY, respectively). This is while the estimates of GCA/SCA ratio as well as broad and narrow sense heritabilities were lower under salinity stress conditions than those obtained for non-saline conditions (Table 4). The GCA/SCA ratios obtained for saline conditions were 0.44 for FWT, 0.26 for NF, and 0.14 for FY. Likewise, the heritabilities calculated (${h}_{n}^{2}$) for these traits in saline conditions were quite low (0.37, 0.17, and 0.12 for FWT, NF, and FY, respectively). However, a higher dominance variance(${\sigma }_{D}^{2}$) than additive variance (${\sigma }_{A}^{2}$) was obtained in saline conditions when compared with those obtained for non-saline conditions (Table 4).

The results of mean comparisons showed significantly declining trends for yield and its components under saline conditions when compared with those under non-saline conditions (Supplementary Table 1). Under non-saline conditions, the hybrids H8 × 11, H6 × 11, and H4 × 11 produced the heaviest fruit weight of 3.07, 3.04, and 2.92 kg, respectively. Under saline conditions, however, H4 × 11 with 2.39 kg proved the superior hybrid for this trait. The highest values of NF were recorded for H1 × 10, H7 × 10, H3 × 10, H2 × 6, P2, and P7 under non-saline conditions as well as P2, H3 × 8, H7 × 10, and H8 × 10 under saline conditions (Supplementary Table 1). The hybrids H4 × 11 (67.17 t ha^−1^), H5 × 6 (63.19 t ha^−1^), H2 × 9 (62.3 t ha^−1^), H1 × 9 (61.72 t ha^−1^), H2 × 6 (59.52 t ha^−1^), and H4 × 5 (59.49 t ha^−1^) displayed the highest FY values in non-saline conditions while H2 × 9 (44.32 t ha^−1^), H4 × 11 (37.3 t ha^−1^), and H4 × 5 (33.92 t ha^−1^) were those with high yielding capacities under saline conditions (Supplementary Table 1).

The parent P11 followed by P9 and P6 recorded the highest GCA estimates for FWT under non-saline conditions, while P11 with the highest GCA value was the best general combiner in saline conditions (Table 5). Combining ability of parents for FY was also affected by salinity stress so that P9 and P4 recorded the highest significant GCA values for FY (5.17 and 4.2 t ha^−1^, respectively) under non-saline conditions, while the highest positive GCAs for this trait under saline conditions belonged to P9 (2.47 t ha^−1^), P5 (1.99 t ha^−1^), and P2 (1.8 t ha^−1^) (Table 5). Finally, the parents P10, for FWT and FY, and P11, for NF, recorded the highest negative GCA values in both experimental conditions.

Results of SCA analysis showed that the highest positive and the highest negative values for FWT in non-saline conditions belonged to H4 × 5 and H1 × 11, respectively (Table 6). Under saline conditions, however, the results were different in that while the hybrid H4 × 11 recorded the highest positive SCA value for FWT, H4 × 6 and H2 × 3 recorded the highest negative values (Table 7). Under non-stress conditions, H1 × 10 and H2 × 11 exhibited the highest positive SCA value but H2 × 3 and H2 × 5 exhibited the highest negative SCA values for NF (Table 6). Under salinity stress, however, H4 × 6 and H3 × 7 recorded the highest positive and negative SCA values for NF, respectively (Table 7).

Based on combining ability analysis of melon hybrids for FY, 28 hybrids recorded significant SCAs under non-saline conditions and 13 under saline conditions (Tables 6 and 7). The highest positive SCA values under non-saline conditions belonged to H4 × 11 (19.19 t ha^−1^), H5 × 6 (17.37 t ha^−1^), and H2 × 9 (15.33 t ha^−1^) while the highest negative ones were recorded for H5 × 9 (−15.75 t ha^−1^) and H4 × 10 (−13.31 t ha^−1^) (Table 6). More or less similar results were obtained for saline conditions as H2 × 9 (16.58 t ha^−1^) and H4 × 11 (12.14 t ha^−1^) exhibited the highest positive SCA values for FY but H7 × 11 (−7.63 t ha^−1^) and H2 × 5 (−7.47 t ha^−1^) recorded the highest negative ones (Table 7).

The heterosis and heterobeltiosis results obtained for the studied traits under non-saline and saline conditions are reported in Supplementary Tables 2 and 3, respectively. On average, the observed heterosis for yield was 10.2% in non-saline conditions, while a heterosis value of 14.6% and a heterobeltiosis value of 6.7% were recorded for yield under salinity stress conditions. Twenty-four F1 hybrids of the non-saline treatment and twenty-eight of the saline treatment displayed heterobeltiosis. Finally, the hybrids H5 × 6, H2 × 6, H4 × 11, and H3 × 11 from the non-saline treatment, and H4 × 11, H4 × 5, H2 × 9, and H3 × 5 from the saline one exhibited the highest heterosis and heterobeltiosis values, respectively, for yield.

### Fruit quality-related traits

Based on combined ANOVA results, fruit quality (both physical and chemical) traits were significantly (*P* < 0.01) affected by salinity stress (Table 3). In addition, the melon genotypes were significantly different (*P* < 0.01) for all the quality traits studied. While genotype × salinity interaction was significant for fruit length (FL), fruit pulp thickness (FT) and total soluble solids content (TSS) (Table 3), SCA and GCA effects were significant for all the traits and indicated that both dominant and additive gene effects were involved in their inheritance. Exception for peel thickness (PT), GCA × salinity interaction was significant for all the traits, while SCA × salinity interaction was only significant for TSS (Table 3).

SCA and GCA were significant for all the traits under both environmental conditions, except for SCA that was only significant for FT under non-saline conditions (Table 4). High GCA/SCA ratios were obtained for fruit width (FW) (0.85), seed cavity width (SCW) (0.89), FT (0.93), and TSS (0.6) but relatively low ones for FL (0.43), SCL (0.38), and PT (0.04) (Table 4). Broad-sense heritability estimates ranged from 0.74 for PT to 0.91 for SCL. The results obtained for GCA/SCA ratio and ${h}_{n}^{2}$ estimates were in line with the above mentioned results in the sense that high values were recorded under non-saline conditions for FW, SCW, FT, and TSS (0.74, 0.74, 0.8, and 0.51, respectively) but low ones for FL, seed cavity length (SCL), and PT (0.38, 0.35 and 0.03, respectively) (Table 4). It is clear from this Table that the GCA/SCA ratios as well as the broad- and narrow-sense heritabilities calculated for these traits under salinity stress conditions were even lower than those obtained for the non-saline treatment. This is evidenced by the high GCA/SCA ratios of 0.8, 0.72, and 0.64 obtained under the salinity stress treatment for SCW, FW, and FT, respectively, and the low estimates obtained for other quality traits (0.05 for PT, 0.21 for FL, 0.29 for SCL, and 0.46 for TSS). Heritability estimates revealed that not only were the ${h}_{b}^{2}$ estimates high for these traits but also that those for fruit quality traits agreed with the GCA/SCA ratios ranging from 0.04 for PT to 0.7 for SCW (Table 4).

Mean comparisons showed significant reductions in FL, FW, SCL, SCW, and FT, but significant increases in PT and TSS due to salinity stress (Supplementary Table 1). The hybrid H10 × 11 showed the highest FL in melon cultivars grown under both experimental- conditions (22.43 and 18.58 cm, respectively). The highest FW values were recorded by H9 × 11 (20.82 cm) and H1 × 9 (20.51 cm) under the non-saline treatment and by H4 × 11 (19.03 cm) in saline conditions. The parents P9 and P8 as well as the hybrid H8 × 9 exhibited the lowest SCL values under both experimental conditions. Regarding SCW, H3 × 8 and P8 recorded the lowest values under non-saline conditions while P3 did so in the saline treatment. The thickest fruit pulp belonged to H9 × 11 in the non-saline treatment and to H4 × 11 in the saline one. This is while the thickest fruit peels were observed in P11, P7, P4, and P3 grown under non-saline conditions but under saline conditions, they were observed in P11, P7, P3, and P6 (Supplementary Table 1). In the non-saline treatment, H8 × 10 and P8 demonstrated the highest TSS, while P6, P8, P3, and H3 × 4 yielded the sweetest fruits under salinity stress (Supplementary Table 1).

Combining ability of the parents revealed that three parents grown under non-saline conditions exhibited significant positive GCAs for FL, with the highest belonging to P6. While under saline conditions, P11 recorded the highest GCA for FL (Table 5). Regarding FW, four parents grown under non-saline conditions had significant positive GCAs with P9 being the best, while in saline conditions, P1 recorded the highest GCA for this trait. These parents can be clearly used to develop new hybrids with larger fruit sizes. Under both experimental conditions, P8 and P10 exhibited the greatest negative GCAs for FL and FW, respectively (Table 5). In non-stress conditions, P6 and P9, and in saline conditions, P10 and P1 recorded the highest positive GCAs for SCL and SCW, respectively (Table 5). This is while P8 grown in non-saline conditions and P9 and P3 grown in saline conditions showed the highest negative GCAs for these same traits. Parent P11 grown in non-saline conditions and P5 grown under salinity stress recorded the highest positive GCAs for FT. The highest GCA for PT was recorded by ‘Samsouri’ (P7) under both treatments. P10 in both environments simultaneously recorded the highest negative GCAs for FT and PT (Table 5). Finally, the combining ability of parents for TSS revealed that four parents grown under non-saline conditions and five under salinity stress had significant positive GCAs, which makes parents potent for improving fruit sweetness. Based on our results, P8 grown in either environment displayed the highest positive GCA (i.e., 0.88 in non-saline conditions and 1.06 under salinity stress) while P9 had the highest negative GCA (−1 and −1.04 under non-saline and salinity stress conditions, respectively) (Table 5).

Based on the combining ability of the hybrids, H10 × 11 and H2 × 10 recorded the highest positive SCAs for FL and H4 × 11 the highest value for FW under both treatments (Tables 6 and 7). However, the highest negative SCA values for FW belonged to H2 × 11 grown under non-saline conditions and to H7 × 10 grown under salinity stress while the highest negative SCAs for FL under both conditions were recorded by H4 × 6.

Negative values of seed cavity size are beneficial. Thus, H1 × 11 and H9 × 10 were identified as promising hybrids for they recorded the highest negative SCA values for SCL, while H5 × 9 and H1 × 11 recorded such values for SCW, both under non-saline conditions (Table 6). Under saline conditions, the highest negative SCA values were measured in H1 × 10 and H9 × 10 for SCL, and in H7 × 9 and H10 × 11 for SCW (Table 7). However, H6 × 7 and H2 × 4 grown under non-saline conditions exhibited significant positive SCA values for FT (Table 6). Under salinity stress, H3 × 7 and H2 × 4 recorded the highest positive SCA values but H1 × 11 obtained the highest negative ones (Table 7). Regarding PT, H7 × 8 displayed the highest positive SCA values in both experimental conditions. For TSS, 8 hybrids grown in either environment showed significant positive SCA values, among which H8 × 10 and H2 × 7 recorded the highest values under both conditions, indicating that they could be used for improving TSS under either condition (Tables 6 and 7).

Favorable heterosis for FL was detected only over mid-parents (5.9%) under non-saline conditions, but over both mid- and best parents (11.8% and 3.8%, respectively) under salinity stress (Supplementary Tables 2 and 3). For FW under either treatment, beneficial heterosis was observed only over the mid-parent (1.2% in non-saline and 5% in saline conditions). Regarding seed cavity size (SCL and SCW), favorable heterosis was not observed under either treatment. Beneficial heterosis was however, detected only based on mid-parent (6.5%) for FT under salinity stress. Fifteen hybrids grown under non-saline conditions and twenty-five grown under salinity stress displayed heterosis over the best parent. H3 × 5 and H3 × 7 in non-saline conditions, and H3 × 7 and H2 × 9 in saline conditions recorded the highest values of both heterosis and heterobeltiosis. Regarding PT, favorable heterosis was not observed in either treatment but H2 × 3 was the best for PT under either treatment. Beneficial heterosis was not observed for TSS in either treatment. However, H5 × 10 and H8 × 10 in non-saline conditions, and H1 × 8 and H9 × 10 in saline conditions displayed the highest heterosis and heterobeltiosis (Supplementary Tables 2 and 3).

## Discussion

Given the complex nature of the genetic architecture of salinity tolerance, knowledge of genetic components of tolerance would be essential for the optimal design of tools and strategies aimed at improving salinity tolerance in crop cultivars^1^. Despite the rather large body of data on genetic analysis under non-saline environmental conditions^17–25^, little has been reported in the literature on the inheritance of salinity tolerance in melon.

The significant GCA and SCA effects on FY and FY components imply that both additive and dominant gene effects are involved in governing these traits in melon. These findings suggest the feasibility of selection schemes for improving yield and its components. The significant genotype × environment (saline vs non-saline) interaction for yield implies that the choice of superior genotypes can be determined by the environment. In addition, the GCA × salinity and SCA × salinity interaction effects on FWT and FY revealed that gene actions were affected by salinity stress and that breeding schemes for improved FWT and, thereby, FY in inbred lines and hybrids should be, therefore, carried out under salinity stress. Our results indicating reduced FWT and NF, as major yield components, are in agreement with those previously reported^6,9,12,13^. In addition, salinity stress was found to be capable of altering the inheritance of these traits, leading to not only lower GCA/SCA ratios but also to reduced broad- and narrow-sense heritability estimates relative to those obtained under non-saline conditions.

The results also revealed the more significant effects of dominant gene actions under saline conditions despite the fact that FWT is mainly governed by additive gene effects under non-saline conditions. These findings imply the superiority of saline environments over non-saline conditions in breeding hybrid cultivars. However, no real comparisons between the two environments can be made since no genetic data been so far reported on salinity-stress conditions. However, in their experiment with non-saline field grown melon, Zalapa *et al*.^19,21^ reported that dominant and epistatic gene effects were involved in controlling FWT while other researchers had highlighted the additive gene effects^17,23^. The narrow-sense heritability obtained for FWT under non-saline conditions was more or less similar to those reported by Feyzian *et al*.^17^, Zalapa *et al*.^21^, and Lippert and Hall^25^, but was much higher than that reported by Pouyesh *et al*.^18^ and Kalb and Davis^23^.

Number of fruits per plant was governed by dominant gene action in both experimental conditions. In contrast to our results, Zalapa *et al*.^21^ reported that additive gene effects controlled this trait but in non-saline conditions; the authors carried out a means analysis on two melon cultivars ‘USDA 846-1’ and ‘TopMark’ grown in two regions. This inconsistency may be attributed to the number of parents, level of genetic diversity, and method of analysis.

Yield is the most important agronomic criterion for tolerance to abiotic stresses including salinity^1^. Salinity stress caused a significant reduction in yield, a finding which is in agreement with those reported elsewhere^6–9^. The GCA/SCA ratio, genetic variance components, and heritability estimates of FY revealed that FY was mostly controlled genetically by dominant gene effects in both non-saline and saline field conditions. These results agree with those reported by Zalapa *et al*.^21^ who found dominant gene action to be the main agent controlling yield per plant. Feyzian *et al*.^17^ and Pouyesh *et al*.^18^ also reported both additive and dominance genetic effects were involved in controlling melon FY under non-saline conditions while Kalb and Davis^23^ claimed that additive gene action was more important in FY genetic control in normally grown melons. In agreement with ${h}_{n}^{2}$ estimates obtained in the current study, some researchers reported low ${h}_{n}^{2}$ estimates for FY in melons grown under non-saline conditions^18,21,25^. This is while Feyzian *et al*.^17^ reported contradictory results (${h}_{n}^{2}$ = 0.6) in their analysis of a melon 7 × 7 diallel, probably because they used melon types (inodorous group) different from those used in the present study.

Despite the availability of reports on the inheritance of agronomic traits under salinity stress, no comprehensive study has yet been conducted on the genetic analysis of these traits in melon under salinity stress. The highly positive values of SCA for yield and its components obtained in the current study revealed that crossed parents offer good combiners, suggesting that hybrid development would be a promising approach, particularly for growing crops in saline environments. Breeding for salinity stress requires best combiners and favorable parents to yield superior hybrids for saline conditions. Given the ultimate goal of plant breeding which is that of improving crop yield under all possible environmental conditions, the parents P9, P4, P6, and P2 as well as H2 × 9 and 4 × 11 may be considered in the development of breeding programs for *C*. *melo* species under both non-saline and saline conditions. The growing global interest in hybridization of melon warrants the acquisition of a basic understanding of the heterosis and quantitative inheritance for yield and other relevant agronomic traits in order to maximize the effectiveness of hybrid cultivars. Similar to variance components and other genetic parameters, heterosis has been reportedly higher in melons grown in saline conditions than in normally grown ones, reflecting the outperformance of melon hybrids under salinity stress. These findings are promising due to the apparent correlations that can be established between the higher values of dominance effects – as one of the mechanisms hypothetically involved in heterosis – and those of heterosis for agronomic traits under salinity stress. Our data clearly show that salinity stress induces certain genes to be expressed in hybrids with non-additive gene expression patterns while the potential impacts of salinity on genetic and epigenetic transcriptional regulation remain largely unclear. This is confirmed by Meyer *et al*.^28^ who demonstrated differentially expressed genes between the parental genotypes and hybrid embryos to suggest that gene regulatory interactions among parental alleles possibly form the genetic mechanism underlying heterosis in maize.

Fruit physical and chemical quality traits were influenced by salinity stress such that fruit size (FL and FW) and pulp percentage decreased but fruit peel thickness and sweetness increased. The significant effects of SCA and GCA for quality traits revealed both dominance and additive gene effects involved in the expression of these traits. The significant interaction of GCA × salinity for all the traits, except for PT, indicates that additive gene effects for these traits depend on the environment (non-saline or saline) in which the genotype grows. Like the FY and FY components, inheritance of quality traits were affected by salinity stress and the estimated parameters were often lower than those obtained under non-saline conditions.

The genetic results obtained for fruit shape and seed cavity size revealed that FL and SCL were controlled by the higher dominance gene effects, while higher additive gene effects were involved in governing FW and SCW, under both conditions. P8 in the non-saline treatment and P9 and P3 in the saline one were found to be the desirable ones for increasing fruit pulp by reducing within-hybrid seed cavity size relative to fruit size.

Flesh thickness is another quality parameter directly correlated with FWT and, thereby, with FY as previously reported^25^. The reduced value for this trait in the current study is consistent with those reported elsewhere on different melon cultivars exposed to salinity stress^12–14^. Under both experimental conditions, FT was governed by the higher impact of additive gene actions. Therefore, selection of this trait should expectedly lead to improved FWT and FY values. Consistent with the current findings, Pouyesh *et al*.^18^ reported that additive gene effects were more important for the genetic control of FT under non-saline conditions. Overall, melon genotypes produced fruits with thicker peels under salinity stress than under non-saline conditions. Contrary to this observation, previously published studies reported no effect of salinity stress on PT^9,13^. The discrepancy may be attributed to differences in the genotypes used and salinity stress intensity. The lower values (close to zero) of genetic parameters indicate that PT is mainly governed by dominance or even over-dominance gene actions.

Sugar content is undoubtedly the most important quality and marketability parameter of melon fruits. Salinity was observed to improve fruit quality as reflected by TSS. In agreement with our results, some researchers reported increased TSS in melon due to exposure to salinity stress^8,9^. Regarding the inheritance of this trait, both additive and dominance gene actions (but with a greater role played by additive effects) were found to be involved in governing sweetness in melons grown under both conditions. In addition, the significant SCA × salinity interaction for TSS indicated that the dominance gene effects could be modified by varying salinity intensity, suggesting that selection of premier hybrids should be accomplished under specifically designed environmental conditions. Pouyesh *et al*.^18^ found additive gene effects to be the major genetic action for TSS under non-saline conditions. Harel-Beja *et al*.^29^ and Monforte *et al*.^27^ found six and five QTLs, respectively, which were governed by additive gene effects for TSS under non-saline conditions. The parents P8, P2, and P1 as well as H8 × 10 and H2 × 7 might have important roles to play in improving the sweetness of melons cultivated in both non-saline and saline conditions.

While the various traits measured differed in the extent to which they were determined by additive (e.g. seed cavity width and fruit flesh thickness) versus dominant (fruit peel thickness) effects under normal conditions, a decline in GCA/SCA ratio for most traits was evident under saline conditions, indicating the increasing importance of dominant effects under this environmental stress. In addition, both GCA and SCA showed a significant interaction with salinity treatment, which was coupled with a reduction in broad sense heritability for a number of traits. The increased phenotypic variance due to environmental factors versus genetic factors under NaCl stress suggests that it would be worthwhile carrying out breeding programs for improved fruit yield and quality under those same conditions to develop hybrids most suitable for growth on saline soils, a globally increasing problem.

Heterosis was also found favorable for improving fruit size (FL and FW) and pulp thickness. However, beneficial heterosis for fruit quality parameters in melon has been previously reported; examples include Monforte *et al*.^30^ for FL and FW; Kalb and Davis^22^ for SCW; Kalb and Davis^22^ and Kitroongruang *et al*.^24^ for FT; and finally, Kalb and Davis^22^, Kitroongruang *et al*.^24^, and Lippert and Hall^25^ for TSS, all grown under non-saline conditions.

## Conclusions

The assessment of yield and yield components seems to be the best approach for improving salinity tolerance in melon and selecting genotypes of high yield and desired quality parameters to be grown under both non-saline and salinity stressed conditions. Salinity stress generally leads to declining melon performance and productivity while it enhances fruit sweetness. Overall, high genetic variations were recorded for all the traits in the melon germplasm grown under field conditions. The observations indicate that the genetic mechanisms underlying these traits were affected by salinity stress to increase the extent of dominance gene actions. It may be concluded that any breeding strategy should rely on hybrid development especially tailored to the target environment. In addition, the magnitude of heterosis for yield-associated and quality traits under both environmental conditions shows the potential for enhancing fruit yield and quality parameters through a systematic search for heterotic groups and evaluation of parents for their specific combining abilities. The findings of the present study might have important implications for understanding the genetic mechanisms involved in salinity tolerance and for improving yield and quality in melon cultivars grown in saline conditions.

## Methods

### Plant material and experimental conditions

**Figure 1:**
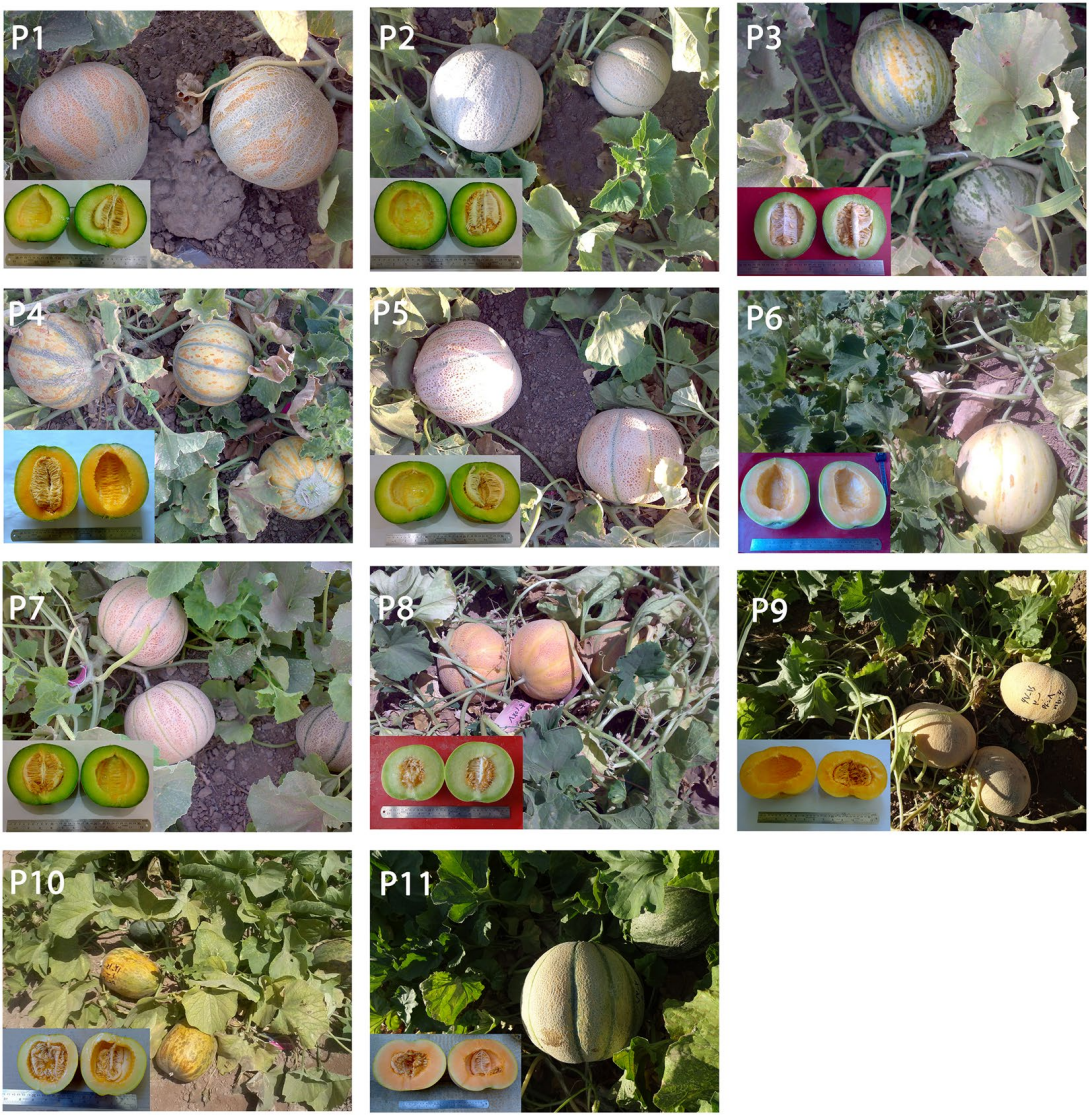
Showing genetic variations especially in fruit characteristics of melon parents used in diallel analysis. (P1–P11; correspond to the parent codes listed in Table 1).

Eleven melon cultivars (parents) with different levels of variability with respect to yield and salinity tolerance were used to develop 55 hybrids according to a half diallel mating design. A preliminary evaluation of Iranian melon cultivars for salinity tolerance was conducted in two growing seasons (2014‒2015) to screen salinity tolerant cultivars^31,32^. The list of parents along with their descriptions is provided in Table 1. Figure 1 presents some of the distinguishing plant and fruit characteristics of the parent genotypes used.

**Table 1:** Description, origin and geographical determinants of melon parents used in this study.

Parental code	Cultivar name	Descriptive features (fruit shape, color and flesh color)	Origin	Latitude	Longitude	Altitude (m)
1	Rishbaba-1	Oblate, pale green, pale green	Badrud, Iran	33°41′N	52°0′E	996
2	Shahabadi-1	Globular, cream, green	Isfahan, Iran	32°40′N	51°42′E	1572
3	Magasi	Elliptical, green, green	Neyshabur, Iran	36°13′N	58°49′E	1224
4	Till-Toroq	Elliptical, orange, salmon	Toroq, Iran	36°12′N	59°39′E	998
5	Savehie	Globular, cream, green	Saveh, Iran	35° 1′N	50°21′E	1008
6	Sabouni	Elliptical, pale green, salmon	Mashhad, Iran	36°18′N	59°34′E	999
7	Samsouri	Globular, cream, green	Varamin, Iran	35°20′N	51°38′E	930
8	Lacki	Oblate, white, pale green	Mashhad, Iran	36°18′N	59°34′E	996
9	Dastjerdi	Flattened, light yellow, orange	Isfahan, Iran	32°40′N	51°42′E	1572
10	Gargar	Pyriform, orange, yellow	Eyvan, Iran	33°49′N	46°18′E	1178
11	Majidi-Abarkouh	Ovate, light yellow, orange	Abarkouh, Iran	31°7′N	53°16′E	1509

**Table 2:** Physico-chemical characteristics of the field experimental soil.

Water-holding capacity at field capacity (g kg^−1^)	Clay (%)	Silt (%)	Sand (%)	EC (dS m^−1^)	pH	Organic matter (%)	Available P (mg kg^−1^)	Available K (mg kg^−1^)	Total N (%)
230	30	30	40	1.26	7.4	0.31	32.4	310	0.053

**Table 3:** Combined analysis of variance for different agronomic and quality related traits in melon parents and hybrids cultivated in non-saline and saline field conditions.

Source of variation	Mean square										
	df	FWT	NF	FY	FL	FW	SCL	SCW	FT	PT	TSS
Salinity (S)	1	21.67^**^	29.52^**^	18171^**^	299^**^	232^**^	264^**^	75.38^**^	14.03^**^	26.58^**^	147^**^
Rep (Salinity)	2	0.10	1.87	194	13.17	2.44	10.10	1.35	0.11	0.04	0.63
Genotype (G)	65	0.77^**^	0.54^**^	223^**^	12.56^**^	12.56^**^	9.99^**^	5.70^**^	0.83^**^	0.39^**^	4.19^**^
G × S	65	0.12^**^	0.13^ns^	68.96^**^	1.13^*^	1.16^ns^	0.66^ns^	0.61^ns^	0.10^*^	0.02^ns^	0.72^**^
GCA	10	3.38^**^	1.36^**^	625^**^	34.60^**^	63.57^**^	27.77^**^	29.93^**^	4.24^**^	0.46^**^	16.22^**^
SCA	55	0.29^**^	0.39^**^	150^**^	8.55^**^	3.29^**^	6.76^**^	1.30^**^	0.21^**^	0.38^**^	2.01^**^
GCA × S	10	0.40^**^	0.14^ns^	176^**^	1.47^*^	2.40^**^	0.99^*^	1.60^**^	0.26^**^	0.03^ns^	1.19^**^
SCA × S	55	0.07^*^	0.13^ns^	49.33^**^	1.07^ns^	0.94^ns^	0.60^ns^	0.43^ns^	0.07^ns^	0.02^ns^	0.63^**^
Residual	130	0.04	0.1	15.35	0.76	0.91	0.52	0.46	0.072	0.04	0.24
CV		13	16.7	12.3	6.3	6.3	8.1	7.8	8	9.4	4.4
R^2^		0.93	0.85	0.95	0.92	0.9	0.93	0.89	0.89	0.9	0.94

FWT; fruit weight; NF, number of fruit; FY, yield; FL, fruit length; FW, fruit width; SCL, seed cavity length; SCW, seed cavity width; FT, fruit flesh thickness; PT, fruit peel thickness; TSS, fruit total soluble solids. ns: non-significant, *Significant at P < 0.05 and ^**^Significant at P < 0.01.

**Table 4:** Analysis of combining ability and estimation of genetic parameters including general (${\sigma }_{GCA}^{2}$) and specific (${\sigma }_{SCA}^{2}$) combining ability variances, additive (${\sigma }_{A}^{2}$) and dominance (${\sigma }_{D}^{2}$) variances, GCA/SCA ratio, broad-sense heritability (${h}_{b}^{2}$) and narrow sense heritability (${h}_{n}^{2}$) of under non-saline and saline field conditions.

Environment	Estimate	FWT	NF	FY	FL	FW	SCL	SCW	FT	PT	TSS
Non-saline	GCA	2.95^**^	0.99^**^	700^**^	23.99^**^	40.97^**^	18.07^**^	19.36^**^	37.46^**^	0.21^**^	7.5^**^
	SCA	0.19^**^	0.31^**^	144^**^	4.77^**^	2.06^**^	4.05^**^	0.99*	0.12^ns^	0.18^**^	0.96^**^
	Error	0.05	0.12	19.54	0.82	1.03	0.57	0.62	0.08	0.05	0.28
	CV	11.5	15.4	11	6.1	6.3	7.6	8.6	8	11.3	5
	R^2^	0.93	0.78	0.92	0.91	0.89	0.92	0.86	0.87	0.79	0.88
	${\sigma }_{GCA}^{2}$	0.11^**^	0.03^**^	21.39^**^	0.74^**^	1.5^**^	0.54^**^	0.71^**^	0.11^**^	0.001^ns^	0.25^**^
	${\sigma }_{SCA}^{2}$	0.07^**^	0.09^**^	62.51^**^	1.98^**^	0.51^**^	1.74^**^	0.18*	0.02^ns^	0.06^**^	0.34^**^
	${\sigma }_{A}^{2}$	0.21	0.05	42.78	1.48	2.99	1.08	1.41	0.23	0.002	0.5
	${\sigma }_{D}^{2}$	0.07	0.09	62.51	1.98	0.51	1.74	0.18	0.02	0.06	0.34
	GCA/SCA	0.75	0.36	0.41	0.43	0.85	0.38	0.89	0.93	0.04	0.6
	${h}_{b}^{2}$	0.92	0.71	0.92	0.89	0.87	0.91	0.84	0.86	0.74	0.86
	${h}_{n}^{2}$	0.69	0.25	0.37	0.38	0.74	0.35	0.74	0.8	0.03	0.51
Saline	GCA	0.83^**^	0.5^**^	102^**^	12.08^**^	25^**^	10.69^**^	12.18^**^	1.42^**^	0.28^**^	9.91^**^
	SCA	0.17^**^	0.21^**^	52.18^**^	4.85^**^	2.17^**^	3.32^**^	0.74^**^	0.17^**^	0.23^**^	1.68^**^
	Error	0.04	0.08	11.16	0.71	0.8	0.48	0.29	0.06	0.04	0.2
	CV	15	18.3	14.2	6.6	6.3	8.7	6.7	8	8	3.8
	R^2^	0.87	0.79	0.86	0.9	0.88	0.91	0.9	0.86	0.85	0.94
	${\sigma }_{GCA}^{2}$	0.03^**^	0.01^*^	1.8^ns^	0.28^**^	0.88^**^	0.28^**^	0.44^**^	0.05^**^	0.002^ns^	0.32^**^
	${\sigma }_{SCA}^{2}$	0.07^**^	0.06^**^	22.01^**^	2.07^**^	0.68^**^	1.42^**^	0.22^**^	0.05^**^	0.09^**^	0.74^**^
	${\sigma }_{A}^{2}$	0.05	0.02	3.61	0.56	1.76	0.57	0.88	0.10	0.004	0.63
	${\sigma }_{D}^{2}$	0.07	0.06	22.01	2.07	0.68	1.42	0.22	0.05	0.09	0.74
	GCA/SCA	0.44	0.26	0.14	0.21	0.72	0.29	0.8	0.64	0.05	0.46
	${h}_{b}^{2}$	0.86	0.67	0.82	0.88	0.86	0.89	0.88	0.83	0.82	0.93
	${h}_{n}^{2}$	0.37	0.17	0.12	0.19	0.62	0.25	0.7	0.53	0.04	0.43

FWT; fruit weight; NF, number of fruit; FY, yield; FL, fruit length; FW, fruit width; SCL, seed cavity length; SCW, seed cavity width; FT, fruit flesh thickness; PT, fruit peel thickness; TSS, fruit total soluble solids. ns: non-significant, *Significant at P < 0.05 and **Significant at P < 0.01.

**Table 5:** Estimated general combining ability (GCA) of the parents in non-saline and salinity stress conditions.

Environment	Parent	FWT	NF	FY	FL	FW	SCL	SCW	FT	PT	TSS
Non-saline	Rishbaba (P1)	0.22^**^	−0.17^*^	2.69^**^	0.07^ns^	1.11^**^	−0.03^ns^	0.85^**^	0.22^**^	−0.07^ns^	0.39^**^
	Shahabadi (P2)	−0.10^*^	0.22^**^	1.73^*^	−0.44^*^	−0.63^**^	−0.13^ns^	−0.60^**^	−0.11^*^	−0.01^ns^	−0.29^**^
	Magasi (P3)	−0.15^**^	0.07^ns^	−2.13^*^	0.92^**^	−0.98^**^	0.80^**^	−0.97^**^	−0.10^nss^	0.01^ns^	0.50^**^
	Till-Toroq (P4)	0.08^ns^	−0.02^ns^	4.2^**^	0.30^ns^	0.13^ns^	0.32^*^	−0.28^ns^	0.22^**^	0.03^ns^	0.10^ns^
	Savehie (P5)	0.01^ns^	0.08^ns^	2.39^**^	−0.64^**^	0.27^ns^	−0.36^*^	0.22^ns^	0.03^ns^	−0.03^ns^	−0.45^**^
	Sabouni (P6)	0.32^**^	−0.10^ns^	3.36^**^	1.50^**^	0.62^**^	1.12^**^	0.29^ns^	0.18^**^	0.01^ns^	−0.13^ns^
	Samsouri (P7)	−0.28^**^	0.26^**^	−3.73^**^	−0.87^**^	−0.69^**^	−0.70^**^	−0.51^**^	−0.16^**^	0.14^**^	−0.13^ns^
	Laki (P8)	−0.3^**^	−0.01^ns^	−6.02^**^	−1.46^**^	−0.84^**^	−1.27^**^	−1.05^**^	0.04^ns^	0.11^**^	0.88^**^
	Dastjerdi (P9)	0.37^**^	−0.23^**^	5.17^**^	−0.82^**^	2.00^**^	−1.25^**^	1.64^**^	0.24^**^	−0.04^ns^	−1.00^**^
	Gargar (P10)	−0.64^**^	0.23^**^	−11.4^**^	0.08^ns^	−2.37^**^	0.72^**^	−0.51^**^	−0.91^**^	−0.20^**^	0.49^**^
	Majidi-Ab (P11)	0.49^**^	−0.33^**^	3.71^**^	1.36^**^	1.38^**^	0.78^**^	0.94^**^	0.35^**^	0.05^ns^	−0.37^**^
	LSD (0.05; g_i_-g_j_)	0.12	0.19	2.45	0.5	0.56	0.42	0.44	0.16	0.12	0.29
Saline	P1	0.17^**^	−0.15^*^	−0.57^ns^	0.01^ns^	1.20^**^	−0.05^ns^	1.13^**^	0.12^*^	−0.11^**^	0.20^*^
	P2	−0.03^ns^	0.13^ns^	1.80^*^	−0.34^ns^	−0.26^ns^	−0.23^ns^	−0.29^**^	0.01^ns^	0.09^*^	−0.28^**^
	P3	−0.05^ns^	0.08^ns^	−0.06^ns^	0.88^**^	−0.89^**^	0.66^**^	−0.86^**^	−0.04^ns^	0.03^ns^	0.79^**^
	P4	0.05^ns^	−0.04^ns^	0.72^ns^	0.29^ns^	0.22^ns^	0.25^ns^	−0.11^ns^	0.10^*^	0.02 ^ns^	0.31^**^
	P5	0.09^*^	−0.02^ns^	1.99^**^	−0.33^ns^	0.53^**^	−0.06^ns^	0.32^**^	0.17^**^	−0.05 ^ns^	−0.69^**^
	P6	0.06^ns^	−0.14^*^	−0.55^ns^	0.76^**^	0.05^ns^	0.45^**^	−0.19^ns^	0.16^**^	0.06 ^ns^	0.29^**^
	P7	−0.17^**^	0.15^*^	−1.36^ns^	−0.62^**^	−0.70^**^	−0.48^**^	−0.58^**^	−0.13^**^	0.14^**^	−0.03^ns^
	P8	−0.12^**^	0.16^*^	−0.75^ns^	−1.05^**^	−0.34^ns^	−0.96^**^	−0.55^**^	0.04^ns^	0.04 ^ns^	1.06^**^
	P9	0.14^**^	−0.07^ns^	2.47^**^	−0.77^**^	1.09^**^	−1.05^**^	0.95^**^	0.08^ns^	−0.05 ^ns^	−1.04^**^
	P10	−0.39^**^	0.14^ns^	−4.64^**^	0.25^ns^	−2.00^**^	0.80^**^	−0.57^**^	−0.65^**^	−0.24^**^	−0.19^*^
	P11	0.25^**^	−0.23^**^	0.96^ns^	0.91^**^	1.10^**^	0.67^**^	0.75^**^	0.14^**^	0.06^ns^	−0.40^**^
	LSD (0.05; g_i_-g_j_)	0.11	0.16	1.85	0.47	0.49	0.38	0.3	0.14	0.11	0.25

FWT; fruit weight; NF, number of fruit; FY, yield; FL, fruit length; FW, fruit width; SCL, seed cavity length; SCW, seed cavity width; FT, fruit flesh thickness; PT, fruit peel thickness; TSS, fruit total soluble solids. ns: non-significant, ^*^Significant at P < 0.05 and ^**^Significant at P < 0.01.

**Table 6:** Estimated specific combining ability (SCA) of the hybrids in non-saline conditions.

Genotype	FWT	NF	FY	FL	FW	SCL	SCW	FT	PT	TSS
H1 × 2	−0.04^ns^	0.04^ns^	−2.66^ns^	−0.37^ns^	−0.12^ns^	−0.55^ns^	−0.05^ns^	0.04^ns^	0.05^ns^	0.18^ns^
H1 × 3	0.15^ns^	0.10^ns^	6.42^*^	−0.46^ns^	0.33^ns^	−0.14^ns^	0.47^ns^	−0.01^ns^	−0.17^ns^	−0.10^ns^
H1 × 4	−0.09^ns^	−0.06^ns^	−4.07^ns^	0.22^ns^	−0.21^ns^	0.54^ns^	−0.75^ns^	0.18^ns^	−0.01^ns^	−0.31^ns^
H1 × 5	0.28^*^	−0.16^ns^	2.19^ns^	0.89^ns^	0.88^ns^	0.03^ns^	0.31^ns^	0.12^ns^	0.06^ns^	−0.08^ns^
H1 × 6	0.27^ns^	−0.08^ns^	−1.99^ns^	0.61^ns^	0.49^ns^	0.67^ns^	0.47^ns^	0.04^ns^	0.07^ns^	0.91^**^
H1 × 7	0.47^**^	−0.34^ns^	4.06^ns^	1.01^ns^	0.67^ns^	1.02^ns^	0.56^ns^	0.10^ns^	0.01^ns^	0.37^ns^
H1 × 8	−0.26^ns^	−0.57^*^	−7.90^**^	−0.10^ns^	−1.29^ns^	−0.12^ns^	−0.96^ns^	−0.23^ns^	−0.12^ns^	−1.09^**^
H1 × 9	−0.02^ns^	0.40^ns^	13.79^**^	−0.24^ns^	1.32^*^	0.10^ns^	1.46^**^	−0.22^ns^	−0.13^ns^	0.10^ns^
H1 × 10	−0.26^ns^	0.73^**^	0.80^ns^	−1.05^ns^	−0.51^ns^	−0.32^ns^	−0.19^ns^	−0.01^ns^	−0.12^ns^	0.14^ns^
H1 × 11	−0.94^**^	0.25^ns^	−9.92^**^	−1.40^ns^	−2.20^**^	−1.91^**^	−1.32^*^	−0.35^ns^	0.22^ns^	−0.96^**^
H2 × 3	−0.21^ns^	−0.91^**^	−11.83^**^	−0.82^ns^	−0.45^ns^	−0.46^ns^	−0.33^ns^	−0.20^ns^	−0.29^*^	−0.05^ns^
H2 × 4	0.09^ns^	0.38^ns^	7.49^*^	0.02^ns^	0.55^ns^	−0.16^ns^	−0.25^ns^	0.42^*^	0.11^ns^	−0.04^ns^
H2 × 5	0.10^ns^	−0.83^**^	−11.61^**^	−0.39^ns^	0.54^ns^	−0.57^ns^	0.15^ns^	0.14^ns^	0.24^ns^	−0.02^ns^
H2 × 6	0.24^ns^	0.59^*^	14.35^**^	0.10^ns^	0.97^ns^	0.23^ns^	0.18^ns^	−0.17^ns^	0.05^ns^	−0.05^ns^
H2 × 7	−0.17^ns^	−0.60^*^	−8.74^**^	−0.55^ns^	−0.09^ns^	−0.23^ns^	0.12^ns^	−0.08^ns^	0.02^ns^	1.31^**^
H2 × 8	0.01^ns^	−0.08^ns^	−1.91^ns^	−0.10^ns^	0.05^ns^	0.46^ns^	0.73^ns^	−0.11^ns^	0.11^ns^	−1.24^**^
H2 × 9	0.21^ns^	0.40^ns^	15.33^**^	0.93^ns^	0.32^ns^	0.18^ns^	−0.44^ns^	0.28^ns^	−0.09^ns^	0.57^ns^
H2 × 10	0.49^**^	−0.39^ns^	7.85^**^	4.45^**^	1.25^ns^	4.42^**^	0.46^ns^	−0.08^ns^	0.07^ns^	−0.31^ns^
H2 × 11	−0.12^ns^	0.73^**^	1.68^ns^	−0.88^ns^	−1.89^**^	−1.04^ns^	−0.41^ns^	−0.07^ns^	−0.33^*^	0.09^ns^
H3 × 4	−0.18^ns^	−0.21^ns^	−8.42^**^	−1.34^*^	−1.12^ns^	−0.96^ns^	−0.46^ns^	−0.27^ns^	0.24^ns^	0.22^ns^
H3 × 5	0.16^ns^	0.35^ns^	7.86^**^	1.51^*^	0.26^ns^	1.06^*^	−1.11^*^	0.36^ns^	−0.18^ns^	0.5^ns^
H3 × 6	−0.19^ns^	−0.43^ns^	−11.15^**^	−0.57^ns^	−0.17^ns^	−0.22^ns^	0.11^ns^	−0.13^ns^	0.21^ns^	−0.65^ns^
H3 × 7	0.14^ns^	0.30^ns^	7.88^**^	1.05^ns^	0.34^ns^	0.65^ns^	0.11^ns^	0.20^ns^	−0.33^*^	−0.10^ns^
H3 × 8	−0.02^ns^	0.44^ns^	6.55^*^	−0.12^ns^	0.04^ns^	0.02^ns^	−0.26^ns^	0.13^ns^	−0.02^ns^	−0.69^*^
H3 × 9	0.40^**^	−0.29^ns^	2.73^ns^	2.02^**^	0.52^ns^	1.40^**^	0.30^ns^	0.13^ns^	−0.18^ns^	0.34^ns^
H3 × 10	−0.02^ns^	0.45^ns^	0.67^ns^	−0.54^ns^	0.72^ns^	−1.07^*^	0.66^ns^	0.04^ns^	−0.09^ns^	0.16^ns^
H3 × 11	0.39^**^	0.04^ns^	11.56^**^	3.00^**^	0.76^ns^	2.59^**^	0.47^ns^	0.28	−0.27^ns^	0.53^ns^
H4 × 5	0.53^**^	0.06^ns^	12.83^**^	1.52^*^	1.32^*^	1.18^*^	0.86^ns^	0.12^ns^	−0.2^ns^	−0.64^ns^
H4 × 6	−0.51^**^	0.62^**^	−0.50^ns^	−2.10^**^	−1.31^ns^	−1.22^*^	−0.39^ns^	−0.21^ns^	−0.05^ns^	−1.00^**^
H4 × 7	−0.08^ns^	−0.02^ns^	−0.79^ns^	0.01^ns^	−0.59^ns^	−0.01^ns^	−0.12^ns^	−0.08^ns^	−0.16^ns^	−1.01^**^
H4 × 8	0.08^ns^	0.01^ns^	2.10^ns^	−0.09^ns^	0.20^ns^	−0.30^ns^	0.05^ns^	−0.03^ns^	−0.22^ns^	0.92^**^
H4 × 9	0.10^ns^	−0.15^ns^	−5.63^ns^	−0.51^ns^	0.62^ns^	−0.45^ns^	−0.36^ns^	0.30^ns^	0.03^ns^	0.81^*^
H4 × 10	−0.25^ns^	−0.66^**^	−13.31^**^	−0.54^ns^	−0.97^ns^	−0.24^ns^	−0.14^ns^	−0.40^*^	−0.36^*^	−0.53^ns^
H4 × 11	0.46^**^	0.10^ns^	19.19^**^	0.92	2.11^**^	1.14^*^	1.77^**^	0.05^ns^	−0.44^**^	0.86^*^
H5 × 6	0.13^ns^	0.48^*^	17.37^**^	−0.03^ns^	0.82^ns^	−0.04^ns^	0.36^ns^	0.16^ns^	0.01^ns^	−0.67^ns^
H5 × 7	−0.31^*^	0.16^ns^	−3.82^ns^	−0.33^ns^	0.10^ns^	0.19^ns^	0.46^ns^	−0.09^ns^	0.01^ns^	−0.11^ns^
H5 × 8	0.26^ns^	−0.03^ns^	4.28^ns^	0.51^ns^	1.17^ns^	0.50^ns^	1.03^ns^	0.02^ns^	0.15^ns^	0.23^ns^
H5 × 9	−0.15^ns^	−0.45^ns^	−15.75^**^	−0.23^ns^	−2.01^**^	0.03^ns^	−1.71^**^	−0.26^ns^	−0.12^ns^	−0.46^ns^
H5 × 10	−0.14^ns^	−0.33^ns^	−7.03^*^	−0.69^ns^	−1.13^ns^	−0.17^ns^	−0.56^ns^	−0.35^ns^	−0.18^ns^	0.88^*^
H5 × 11	−0.25^ns^	0.25^ns^	2.26^ns^	−0.34^ns^	−0.54^ns^	−0.46^ns^	0.32^ns^	0.33^ns^	−0.08^ns^	−0.26^ns^
H6 × 7	0.37^*^	0.11^ns^	0.65^ns^	2.00^**^	1.37^*^	1.12^*^	0.34^ns^	0.43^*^	−0.18^ns^	−0.65^ns^
H6 × 8	0.38^**^	−0.47^*^	1.84^ns^	1.80^**^	1.57^*^	0.90^ns^	1.09^*^	0.18^ns^	−0.08^ns^	−0.44^ns^
H6 × 9	−0.30^*^	0.22^ns^	0.48^ns^	−0.91^ns^	−1.06^ns^	−0.40^ns^	0.02^ns^	−0.54^**^	−0.24^ns^	−0.89^*^
H6 × 10	−0.26^ns^	−0.35^ns^	−7.30^*^	0.02^ns^	−0.81^ns^	0.18^ns^	−0.36^ns^	0.22^ns^	−0.13^ns^	−0.27^ns^
H6 × 11	0.34^*^	0.02^ns^	9.78^**^	−0.74^ns^	0.25^ns^	−1.01^ns^	−0.52^ns^	0.13^ns^	−0.01^ns^	0.81^*^
H7 × 8	−0.10^ns^	0.23^ns^	0.60^ns^	−0.13^ns^	−0.17^ns^	−0.23^ns^	−0.29^ns^	−0.01^ns^	0.31^*^	0.66^ns^
H7 × 9	0.29^*^	−0.08^ns^	6.11^*^	0.15^ns^	0.88^ns^	0.06^ns^	0.95^ns^	0.04^ns^	−0.24^ns^	−0.81^*^
H7 × 10	−0.17^ns^	0.30^ns^	0.83^ns^	−1.41^*^	−1.44^*^	−0.98^ns^	−0.92^ns^	−0.19^ns^	−0.3^*^	−0.65^ns^
H7 × 11	−0.20^ns^	−0.42^ns^	−6.58^*^	−1.57^*^	−0.40^ns^	−1.06^*^	−0.30^ns^	−0.07^ns^	−0.43^**^	−0.64^ns^
H8 × 9	0.17^ns^	−0.22^ns^	−1.12^ns^	0.65^ns^	0.10^ns^	0.27^ns^	0.35^ns^	0.24^ns^	0.08^ns^	−0.44^ns^
H8 × 10	−0.04^ns^	0.10^ns^	0.82^ns^	−0.34^ns^	−0.70^ns^	0.24^ns^	−0.30^ns^	−0.20^ns^	0.01^ns^	1.45^**^
H8 × 11	−0.33^*^	−0.05^ns^	−7.83^**^	−1.78^**^	−0.92^ns^	−1.38^*^	−1.22^*^	−0.25^ns^	−0.14^ns^	−0.37^ns^
H9 × 10	−0.23^ns^	0.25^ns^	−0.93^ns^	−2.00^**^	−0.08^ns^	−1.60^**^	0.42^ns^	−0.22^ns^	−0.16^ns^	0.22^ns^
H9 × 11	0.33^*^	−0.28^ns^	−3.86^ns^	0.56^ns^	1.36^*^	0.37^ns^	0.79^ns^	0.25^ns^	−0.22^ns^	−0.17^ns^
H10 × 11	0.33^*^	−0.43^ns^	0.84^ns^	6.16^**^	0.20^ns^	6.14^**^	−0.40^ns^	−0.02^ns^	−0.16^ns^	0.46^ns^
LSD (S_ij_-S_ik_)^†^	0.42	0.67	8.48	1.73	1.95	1.45	1.52	0.50	0.42	1.01
LSD (S_ij_-S_kl_)^‡^	0.40	0.64	8.12	1.66	1.87	1.39	1.45	0.53	0.40	0.97

FWT; fruit weight; NF, number of fruit; FY, yield; FL, fruit length; FW, fruit width; SCL, seed cavity length; SCW, seed cavity width; FT, fruit flesh thickness; PT, fruit peel thickness; TSS, fruit total soluble solids. ns: non-significant, *Significant at P < 0.05 and **Significant at P < 0.01. †Least significant difference of SCA for comparing half-sib hybrids and ^‡^Least significant difference of SCA for comparing different hybrids.

**Table 7:** Estimated specific combining ability (SCA) of the hybrids in saline conditions.

Genotype	FWT	NF	FY	FL	FW	SCL	SCW	FT	PT	TSS
H1 × 2	0.02^ns^	0.3^ns^	2.53^ns^	−0.5^ns^	−0.12^ns^	−0.61^ns^	−0.17^ns^	−0.01^ns^	0.08^ns^	−0.05^ns^
H1 × 3	0.51^**^	−0.51^*^	1.16^ns^	2.44^**^	2.06^**^	2.40^**^	1.35^**^	0.38^*^	−0.12^ns^	−0.72^*^
H1 × 4	0.10^ns^	−0.01^ns^	0.26^ns^	0.68^ns^	0.75^ns^	0.56^ns^	0.72^ns^	0.10^ns^	0.08^ns^	−1.14^**^
H1 × 5	−0.28^*^	0.01^ns^	0.75^ns^	−0.17^ns^	−0.62^ns^	0.41^ns^	−0.34^ns^	0.36^*^	0.07^ns^	−0.23^ns^
H1 × 6	−0.40^**^	0.18^ns^	−1.00^ns^	−1.54^*^	−1.06^ns^	−1.10^*^	−0.60^ns^	−0.43^*^	−0.05^ns^	1.46^**^
H1 × 7	0.20^ns^	0.02^ns^	−0.90^ns^	0.53^ns^	0.29^ns^	0.52^ns^	−0.04^ns^	0.16^ns^	−0.09^ns^	0.73^*^
H1 × 8	−0.12^ns^	−0.27^ns^	−3.03^ns^	0.13^ns^	−0.53^ns^	−0.54^ns^	−0.57^ns^	−0.08^ns^	−0.16^ns^	0.88^**^
H1 × 9	0.02^ns^	0.27^ns^	2.85^ns^	0.88^ns^	−0.08^ns^	1.13^*^	−0.33^ns^	0.08^ns^	−0.10^ns^	−0.30^ns^
H1 × 10	−0.39^**^	0.28^ns^	−3.12^ns^	−1.78^**^	−1.24^*^	−1.52^**^	−0.61^ns^	−0.44^**^	−0.24^ns^	−0.27^ns^
H1 × 11	−0.24^ns^	−0.20^ns^	−6.99^**^	−1.37^*^	−0.89^ns^	−0.91^ns^	0.05^ns^	−0.53^**^	0.18^ns^	0.03^ns^
H2 × 3	−0.46^**^	−0.44^ns^	−4.69^ns^	−1.25^ns^	−1.28^*^	−0.97^ns^	−0.56^ns^	−0.45^**^	−0.15^ns^	−0.88^**^
H2 × 4	−0.08^ns^	−0.29^ns^	−6.72^*^	−1.22^ns^	0.07^ns^	−1.08^*^	−0.25^ns^	0.18^ns^	0.03^ns^	0.55^ns^
H2 × 5	−0.18^ns^	−0.34^ns^	−7.47^**^	−0.77^ns^	−0.40^ns^	−0.32^ns^	−0.11^ns^	−0.15^ns^	0.20^ns^	−0.09^ns^
H2 × 6	0.08^ns^	−0.31^ns^	−5.53^*^	0.11^ns^	0.18^ns^	0.38^ns^	0.21^ns^	−0.12^ns^	−0.20^ns^	−0.32^ns^
H2 × 7	−0.15^ns^	−0.06^ns^	0.60^ns^	−0.90^ns^	−0.58^ns^	−0.69^ns^	−0.36^ns^	−0.12^ns^	0.36^**^	0.52^ns^
H2 × 8	0.12^nns^	−0.55^*^	−3.31^ns^	0.50^ns^	0.68^ns^	0.22^ns^	0.39^ns^	0.29^ns^	−0.01^ns^	0.27^ns^
H2 × 9	0.52^**^	0.37^ns^	16.58^**^	1.91^**^	1.73^**^	0.89^ns^	0.80^*^	0.50^**^	−0.15^ns^	0.73^*^
H2 × 10	0.41^**^	−0.52^*^	0.23^ns^	4.65^**^	0.97^ns^	4.36^**^	0.78^*^	0.10^ns^	−0.07^ns^	−0.69^*^
H2 × 11	0.22^ns^	0.27^ns^	5.14^ns^	0.15^ns^	0.64^ns^	−0.11^ns^	0.50^ns^	0.20^ns^	−0.29^*^	−0.25
H3 × 4	−0.33^**^	0.10^ns^	−5.69^*^	−1.17^ns^	−1.43^*^	−0.80^ns^	−0.56^ns^	−0.38^*^	0.52^**^	1.60^**^
H3 × 5	0.49^**^	0.28^ns^	4.99^ns^	1.50^*^	0.01^ns^	0.41^ns^	−0.60^ns^	0.16^ns^	−0.15^ns^	0.32^ns^
H3 × 6	−0.12^ns^	−0.26^ns^	−1.17^ns^	−0.90^ns^	−0.12^ns^	−0.45^ns^	0.13^ns^	−0.11^ns^	0.11^ns^	−0.26^ns^
H3 × 7	0.37^**^	−0.60^*^	−1.38^ns^	2.50^**^	1.43^*^	1.85^**^	−0.02^ns^	0.64^**^	−0.29^*^	0.01^ns^
H3 × 8	−0.04^ns^	0.59^*^	6.71^*^	0.23^ns^	−0.19^ns^	0.04^ns^	−0.52^ns^	0.22^ns^	0.01^ns^	−1.69^**^
H3 × 9	0.38^**^	−0.16^ns^	4.04^ns^	0.40^ns^	1.74^**^	0.47^ns^	1.14^**^	0.19^ns^	−0.24^ns^	−0.13^ns^
H3 × 10	−0.12^ns^	0.32^ns^	−2.01^ns^	−0.80^ns^	0.15^ns^	−0.76^ns^	0.25^ns^	0.11^ns^	−0.07^ns^	−0.12^ns^
H3 × 11	0.13^ns^	0.20^ns^	6.44^*^	1.36^*^	0.08^ns^	0.46^ns^	−0.19^ns^	0.30^ns^	−0.39^**^	−0.59^ns^
H4 × 5	0.17^ns^	0.24^ns^	7.73^**^	1.44^*^	0.57^ns^	1.01^ns^	0.59^ns^	−0.10^ns^	−0.26^ns^	−1.23^**^
H4 × 6	−0.46^**^	0.69^**^	−1.77^ns^	−2.03^**^	−1.18^ns^	−1.11^*^	−0.45^ns^	−0.21^ns^	−0.16^ns^	−1.09^**^
H4 × 7	0.09^ns^	0.01^ns^	4.18^ns^	0.24^ns^	0.12^ns^	−0.10^ns^	0.25^ns^	−0.14^ns^	−0.20^ns^	−0.55^ns^
H4 × 8	0.33^*^	0.21^ns^	5.87^*^	0.83^ns^	0.92^ns^	0.49^ns^	0.56^ns^	0.32^ns^	−0.23^ns^	−0.67^*^
H4 × 9	−0.02^ns^	−0.12^ns^	−2.66^ns^	−1.33^*^	0.53^ns^	−0.73^ns^	−0.43^ns^	0.39^*^	−0.11^ns^	0.40^ns^
H4 × 10	−0.04^ns^	−0.43^ns^	1.02^ns^	1.03^ns^	−0.61^ns^	1.02^ns^	−0.20^ns^	−0.24^ns^	−0.43^**^	−0.88^**^
H4 × 11	0.78^**^	0.01^ns^	12.14^**^	1.87^**^	3.51^**^	1.28^*^	1.63^ns^	0.01^ns^	−0.50^**^	−0.49^ns^
H5 × 6	0.40^**^	−0.12^ns^	4.00^ns^	1.62^*^	1.22^*^	0.54	0.64^ns^	0.20^ns^	−0.04^ns^	−1.16^**^
H5 × 7	0.01^ns^	−0.11^ns^	−2.60^ns^	−0.20^ns^	0.07^ns^	−0.25^ns^	0.09^ns^	0.04^ns^	0.11^ns^	0.09^ns^
H5 × 8	0.15^ns^	−0.09^ns^	2.57^ns^	−0.08^ns^	1.02^ns^	0.12^ns^	0.73^*^	0.11^ns^	−0.09^ns^	−0.66^*^
H5 × 9	−0.02^ns^	−0.20^ns^	2.18^ns^	1.71^*^	−0.18^ns^	0.53^ns^	−0.18^ns^	−0.02^ns^	−0.06^ns^	−0.65^*^
H5 × 10	−0.06^ns^	0.22^ns^	0.84^ns^	−0.22^ns^	−0.11^ns^	−0.31^ns^	−0.08^ns^	0.11^ns^	−0.06^ns^	0.86^**^
H5 × 11	−0.06^ns^	0.34^ns^	3.90^ns^	−0.91^ns^	0.14^ns^	−0.92^ns^	0.18^ns^	0.01^ns^	−0.19^ns^	−0.13^ns^
H6 × 7	0.26^ns^	0.16^ns^	7.85^**^	0.82^ns^	0.42^ns^	0.53^ns^	0.07^ns^	0.28^ns^	−0.36^**^	−1.06^**^
H6 × 8	0.22^ns^	−0.26^ns^	1.67^ns^	0.57^ns^	0.91^ns^	0.27^ns^	0.59^ns^	0.19^ns^	−0.20^ns^	−1.24^**^
H6 × 9	−0.09^ns^	0.01^ns^	−3.19^ns^	0.21^ns^	−0.33^ns^	0.16^ns^	−0.06^ns^	−0.09^ns^	−0.25^ns^	−0.51^ns^
H6 × 10	−0.18^ns^	0.03^ns^	−3.57^ns^	0.24^ns^	−0.42^ns^	0.21^ns^	−0.17^ns^	−0.13^ns^	−0.03^ns^	−0.70^*^
H6 × 11	0.13^ns^	−0.16^ns^	1.89^ns^	0.02^ns^	0.11^ns^	0.10^ns^	−0.27^ns^	0.25^ns^	−0.30^*^	−0.09^ns^
H7 × 8	−0.13^ns^	0.08^ns^	−1.10^ns^	−0.47^ns^	−0.45^ns^	−0.16^ns^	−0.12^ns^	−0.20^ns^	0.29^*^	0.84^**^
H7 × 9	−0.03^ns^	0.30^ns^	2.71^ns^	0.49	0.67^ns^	0.35^ns^	1.06^**^	−0.09^ns^	−0.36^**^	−0.92^**^
H7 × 10	−0.17^ns^	0.55^*^	−1.50^ns^	−0.95	−1.34^*^	−0.73^ns^	−0.98^**^	−0.31^ns^	−0.25^ns^	−1.09^**^
H7 × 11	−0.34^*^	−0.22^ns^	−7.63^**^	−1.14^ns^	−0.19^ns^	−0.65^ns^	−0.26^ns^	−0.19^ns^	−0.49^**^	−0.47^ns^
H8 × 9	−0.04^ns^	−0.29^ns^	−3.91^ns^	−0.40^ns^	−0.52^ns^	−0.43^ns^	−0.23^ns^	−0.01^ns^	0.18^ns^	−0.75^*^
H8 × 10	0.21^ns^	0.37^ns^	2.69^ns^	0.71^ns^	0.35^ns^	0.72^ns^	−0.21^ns^	0.12^ns^	0.05^ns^	0.87^**^
H8 × 11	−0.27^*^	0.15^ns^	−4.03^ns^	−1.06^ns^	−0.81^ns^	−0.76^ns^	−0.35^ns^	−0.23^ns^	−0.28^*^	0.70^*^
H9 × 10	−0.05^ns^	−0.18^ns^	−3.71^ns^	−1.01^ns^	0.19^ns^	−1.29^*^	0.01^ns^	0.12^ns^	−0.12^ns^	1.03^**^
H9 × 11	0.06^ns^	−0.22^ns^	−0.51^ns^	−0.35^ns^	−0.09^ns^	−0.10^ns^	0.11^ns^	−0.12^ns^	−0.05^ns^	−0.17^ns^
H10 × 11	0.22^ns^	−0.28^ns^	1.61^ns^	4.71^**^	−0.64^ns^	4.7^**^	−0.76^*^	−0.06^ns^	−0.15^ns^	0.15^ns^
LSD (S_ij_-S_ik_)^†^	0.38	0.55	6.41	1.61	1.71	1.32	1.04	0.47	0.39	0.86
LSD (S_ij_-S_kl_) ^‡^	0.36	0.53	6.14	1.54	1.64	1.27	0.99	0.45	0.37	0.83

FWT; fruit weight; NF, number of fruit; FY, yield; FL, fruit length; FW, fruit width; SCL, seed cavity length; SCW, seed cavity width; FT, fruit flesh thickness; PT, fruit peel thickness; TSS, fruit total soluble solids. ns: non-significant, *Significant at P < 0.05 and ^**^Significant at P < 0.01. ^†^Least significant difference of SCA for comparing half-sib hybrids and ^‡^Least significant difference of SCA for comparing different hybrids.

**Figure 2:**
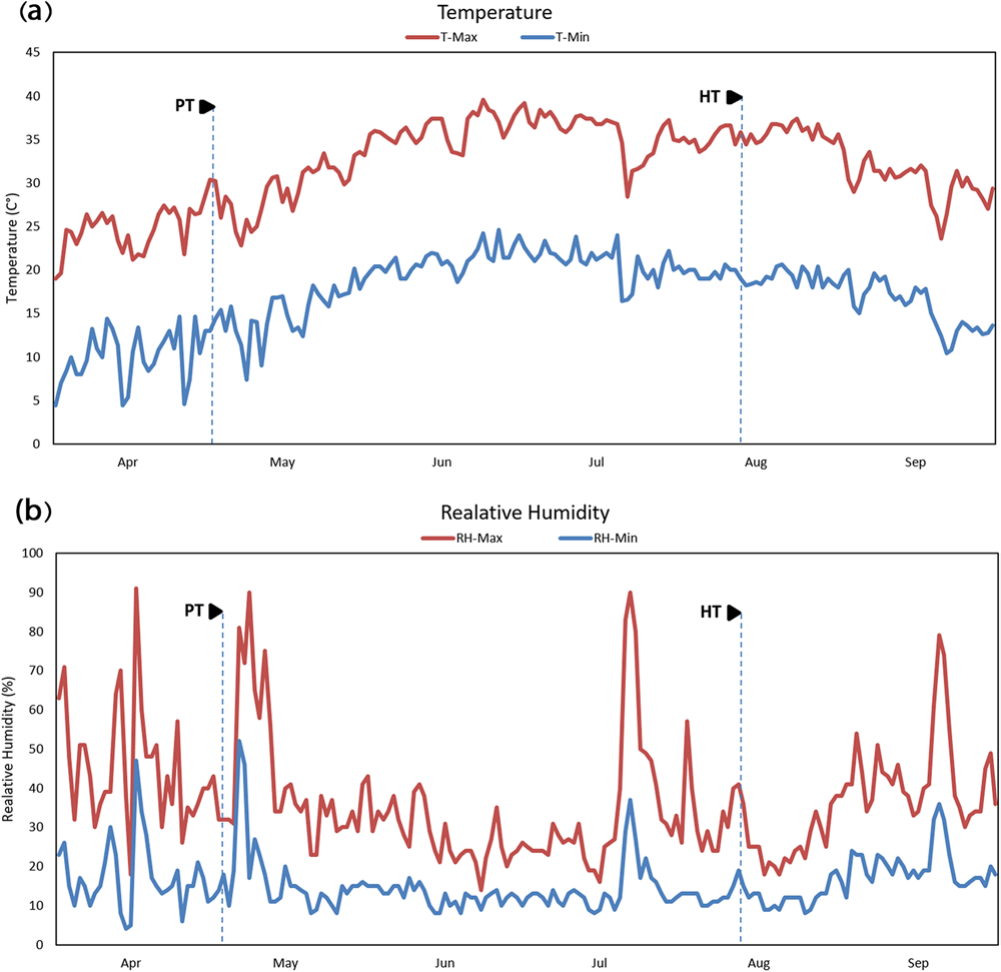
The minimum and maximum (T-Min and T-Max) of daily air temperature (**a**) and minimum (RH-Min) and maximum (RH-Max) relative air humidity (**b**) between April to September 2016, PT and HT corresponded to planting time (3 May) and end of the harvesting time (6 August), respectively.

For the purposes of this study, an open field experiment was carried out in the growing season 2016 (3 May to 6 August) at Lavark Research Station (40 km southwest of Isfahan, latitude 32°32′N, longitude 51°23′E, altitude 1630 m), College of Agriculture, Isfahan University of Technology, Isfahan, Iran. The area is characterized by hot dry summers (average daily minimum and maximum temperatures of 19.5 °C and 35.5 °C, respectively) with almost no precipitation. Mean annual precipitation and mean annual temperature during the experimental period were 140 mm and 14.5 °C, respectively. Figure 2 shows the temperature and relative humidity of the site over the experimental period. The soil is characterized by a silty clay loam texture and is a typic Haplargids of the arid tropic. Table 2 reports the physico-chemical properties of the soil. The electrical conductivity (EC_e_) of saturated-soil extract was determined using a conductivity meter (inoLab® Cond 7110, WTW, Germany).

The parents (11 genotypes) and their 55 F_1_ hybrids along with six local cultivars used as checks were evaluated using a simple rectangular lattice design (8 × 9) with two replications for each of the experimental conditions (saline and non-saline). Each plot consisted of three rows, 6 m long, 3 m spaces between the rows, and 0.5 m spacing between plants within each row. The seeds of the genotypes were planted directly on top of the furrows. Irrigation was applied by furrow irrigation using a schedule commonly used for melon production in the region. In the saline experiment, freshwater was applied until three weeks post-emergence but later shifted to saline water (EC_w_ = 14 dS m^−1^). Identical irrigation volumes, monitored by flow meters, (each irrigation volume equal to 260 m^3^.ha^−1^) were applied to the experimental (both saline and non-saline) plots. The saline treatment irrigated seven times with dissolved sodium chloride (1 M NaCl) flowed from a NaCl supply tank (4000 L) into the irrigation water to maintain the desired level of EC_w_ (14 dS m^−1^) using calibrated flow meters^32^. The EC_e_ was measured in all plots from the 0 to 40 centimeter soil depth at planting and harvesting stages. Using one soil sample from each plot at harvest, average values of EC_e_ for the non-saline and saline field conditions were measured to be of 1.5 and 8.65 dS m^−1^, respectively.

### Measurement of traits

Melon fruits were harvested thrice a week from both experimental plots at the desirable ripening stage and labeled. Harvested melon fruits were counted as the number of fruits per plant (NF). The total number of fruits per plant divided by the total fruit weight per plant was used for the FWT calculation. In addition, FY was measured separately for each plot. Moreover, FL and FW as well as SCL and SCW were determined using the harvested fruits. Radial cross sections of fruits for each plant were used to calculate FT and PT as NF divided by total FT and total PT, respectively. Fruit sweetness, based on total fruit soluble solid content (TSS), was measured using the juice extract obtained from liquefying the mesocarp of each fruit by a portable refractometer (K-0032, Japan) and shown as the °Brix index at 20 °C.

### Data analysis

Prior to analysis of variance (ANOVA), the data were checked for normality of distribution and homogeneity of residuals using Kolmorov-Smirnof and Bartlett’s tests, respectively. The relative efficiency of the rectangular lattice design relative to the randomized complete block design (RCBD) was calculated to be less than 100% for all the traits; hence, the statistical analysis was carried out using RCBD. Mean comparisons were conducted with Fisher LSD test (P < 0.05). The combined ANOVA was performed for environment (salinity), GCA, SCA, and their interactions using the following statistical model:

$${X}_{ijkl}=u+{e}_{i}+{r}_{j(i)}+{g}_{k}+{g}_{l}+{s}_{kl}+e{g}_{ik}+e{g}_{il}+e{s}_{ikl}+{\varepsilon }_{ijkl}$$

where, ${X}_{ijkl}$ is the *ijkl*th observation; *u* is population mean; *g*_*k*_ and *g*_*l*_ are the GCA effects of parent *k*th and *l*th, respectively; *s*_*kl*_ is the SCA value of hybrid involving *k*th and *l*th parents; *eg*_*ik*_, *eg*_*il*_ and *es*_*ikl*_ are the interaction effects between *i*th environment with GCA value of *k*th parent, GCA value of *l*th parent and SCA value of *kl*th hybrid, respectively; and, *ε*_*ijkl*_ is the residual (error) factor.

The analysis of GCA and SCA variances were performed based on Method 2 of Model 1 recommended by Griffing^33^ using the following statistical model:

$${x}_{ij}=u+{g}_{i}+{g}_{j}+{s}_{ij}+\frac{1}{bc}\sum _{k}\,\sum _{l}{e}_{ijkl}$$

where, *u* is the population mean; *g*_*i*_ and *g*_*j*_ are the GCA effects of parent *i*th and *j*th, respectively; *s*_*ij*_ is the SCA effects of the hybrid *i*th × *j*th; and *e*_*ijkl*_ is the residual (error) factor.

The diallel data were analyzed using DIALLEL-SAS program developed by Zhang *et al*.^34^. Accordingly, ${\sigma }_{SCA}^{2}$ and ${\sigma }_{GCA}^{2}$ estimates as well as their variances were calculated for the random-effects model to compute dominance variance (${\sigma }_{D}^{2}$), additive variance (${\sigma }_{A}^{2}$), and heritability (*h*^2^)^34^. Broad-sense (${h}_{b}^{2}$) and narrow-sense (${h}_{n}^{2}$) heritabilities were estimated using the following formulae:

1 $${h}_{b}^{2}=\frac{{\sigma }_{A}^{2}+{\sigma }_{D}^{2}}{{\sigma }_{A}^{2}+{\sigma }_{D}^{2}+\frac{{\sigma }_{E}^{2}}{r}}$$

2 $${h}_{n}^{2}=\frac{{\sigma }_{A}^{2}}{{\sigma }_{A}^{2}+{\sigma }_{D}^{2}+\frac{{\sigma }_{E}^{2}}{r}}$$

Genetic ratio (GCA/SCA ratio), indicating the proportions of additive and dominance effects in the genetic control of traits, was estimated using the following formula^35^:

3 $${\rm{GCA}}/{\rm{SCA}}=\frac{2{\sigma }_{GCA}^{2}}{2{\sigma }_{GCA}^{2}+{\sigma }_{SCA}^{2}}$$

Heterosis (hybrid superiority to parent’s mean; MP) and heterobeltiosis (hybrid superiority to the best parent; BP) were calculated using the formulae given by Fonseca and Patterson^36^:

4 $$Heterosis=\frac{{\overline{F}}_{1}-MP}{MP}\times 100$$

5 $$Heterobeltiosis=\frac{{\overline{F}}_{1}-BP}{BP}\times 100$$

Finally, the least significant differences were also measured for statistical assessment of heterosis (MP) = $\sqrt{3Me/2r}\times t$ and heterobeltiosis (BP) = $\sqrt{2Me/r}\times t$^37^. In these equations, *r* represents the number of replications, *Me* is the mean square of the error, and *t* is the table value of t-student’s at 0.05 or 0.01 probability level.

## Supplementary information

Supplementary information

Dataset 1

## Data Availability

All data used for the analyses presented in this manuscript are available as online supplementary information.
