# Supplementary material for: Inheritance of fruit yield and quality in melon (Cucumis melo L.) grown under field salinity stress
Source: Sci Rep. 2019 May 10;9:7249. doi: 10.1038/s41598-019-43616-6 (PMC6510772; doi:10.1038/s41598-019-43616-6)
Supplement: Supplementary file 1 — Supplementary information [file 41598_2019_43616_MOESM1_ESM.pdf]

Supplementary information

Inheritance of fruit yield and quality in melon (*Cucumis melo* L.) grown under field salinity stress

Mahmoud Akrami* and Ahmad Arzani

Department of Agronomy and Plant Breeding, College of Agriculture, Isfahan University of Technology, Isfahan, 84156-83111, Iran

*Corresponding author E-mail: mahmoud.akrami@ag.iut.ac.ir

Genotype	FWT		NF		FY		FL		FW		SCL		SCW		FT		PT		TSS	
	N	S	N	S	N	S	N	S	N	S	N	S	N	S	N	S	N	S	N	S
Rishbaba (P1)	2.55	1.96	1.75	1.21	45.10	26.07	15.44	13.07	18.62	17.31	9.88	7.64	10.84	10.63	4.18	3.53	1.85	2.52	11.63	12.10
H1×2	1.96	1.49	2.34	1.86	41.83	27.23	14.10	11.88	16.43	15.03	9.19	7.03	9.35	8.76	3.71	3.22	1.89	2.62	10.70	11.77
H1×3	2.11	1.95	2.25	1.00	47.06	24.00	15.38	16.04	16.54	16.58	10.54	10.92	9.51	9.71	3.67	3.56	1.69	2.36	11.20	12.17
H1×4	2.11	1.64	2.00	1.38	42.89	23.88	15.44	13.69	17.11	16.37	10.73	8.67	8.98	9.83	4.18	3.42	1.88	2.55	10.59	11.27
H1×5	2.40	1.30	2.00	1.51	47.34	25.63	15.17	12.21	18.33	15.32	9.54	8.21	10.54	9.21	3.94	3.75	1.88	2.47	10.27	11.18
H1×6	2.71	1.16	1.90	1.47	44.13	21.35	17.04	11.94	18.30	14.39	11.66	7.21	10.76	8.43	4.00	2.95	1.93	2.46	11.58	13.85
H1×7	2.30	1.52	2.00	1.60	43.09	20.64	15.05	12.63	17.17	14.99	10.20	7.90	10.04	8.60	3.73	3.25	2.00	2.50	11.04	12.80
H1×8	1.56	1.25	1.50	1.33	28.84	19.12	13.35	11.80	15.06	14.53	8.48	6.35	8.00	8.11	3.60	3.19	1.84	2.33	10.60	14.04
H1×9	2.46	1.65	2.25	1.63	61.72	28.22	13.85	12.82	20.51	16.42	9.33	7.95	13.11	9.84	3.81	3.38	1.68	2.30	9.90	10.76
H1×10	1.21	.71	3.04	1.85	32.18	15.15	13.94	11.19	14.31	12.17	10.28	7.14	9.30	8.04	2.87	2.14	1.54	1.97	11.43	11.64
H1×11	1.66	1.50	2.00	1.00	36.54	16.88	14.88	12.25	16.38	15.60	8.75	7.62	9.63	10.02	3.79	2.83	2.12	2.69	9.48	11.71
Shahabadi (P2)	1.38	1.01	3.03	2.63	38.56	28.39	12.77	10.70	14.25	12.74	8.51	6.41	7.86	6.89	3.25	2.90	1.94	2.82	9.95	11.43
H2×3	1.43	.78	1.63	1.34	27.85	20.52	14.50	12.00	14.03	11.77	10.13	7.37	7.25	6.38	3.15	2.62	1.63	2.52	10.58	11.53
H2×4	1.96	1.26	2.83	1.38	53.49	19.28	14.73	11.45	16.12	14.24	9.94	6.85	8.03	7.44	4.09	3.38	2.05	2.69	10.19	12.49
H2×5	1.89	1.20	1.72	1.35	32.59	19.79	13.37	11.27	16.26	14.08	8.84	7.30	8.93	8.01	3.62	3.13	2.13	2.79	9.66	10.84
H2×6	2.35	1.44	2.95	1.25	59.52	19.20	16.01	13.25	17.04	14.17	11.13	8.51	9.02	7.82	3.47	3.15	1.96	2.50	9.94	11.60
H2×7	1.34	.97	2.13	1.80	29.34	24.51	12.98	10.86	14.68	12.67	8.85	6.51	8.15	6.86	3.20	2.85	2.07	3.15	11.31	12.12
H2×8	1.50	1.29	2.38	1.32	33.87	21.21	12.84	11.83	14.66	14.28	8.96	6.94	8.24	7.65	3.38	3.44	2.13	2.68	9.77	12.95
H2×9	2.37	1.96	2.64	2.00	62.30	44.32	14.51	13.51	17.77	16.77	8.70	7.53	9.75	9.55	3.96	3.68	1.78	2.45	9.70	11.31
H2×10	1.65	1.32	2.30	1.32	38.28	20.87	18.93	17.28	14.33	12.91	14.92	12.84	8.50	8.01	2.46	2.55	1.79	2.33	10.30	10.74
H2×11	2.16	1.77	2.86	1.75	47.19	31.38	14.88	13.43	14.95	15.68	9.53	8.24	9.09	9.05	3.73	3.44	1.64	2.41	9.17	10.96
Magasi (P3)	1.29	.81	2.48	1.99	29.69	19.16	14.81	12.31	13.52	11.20	10.07	7.90	7.23	6.15	3.10	2.49	2.48	3.01	11.32	14.71
H3×4	1.64	.98	2.09	1.73	33.73	18.45	14.72	12.72	14.11	12.11	10.07	8.02	7.45	6.55	3.41	2.78	2.20	3.12	11.23	14.61
H3×5	1.90	1.84	2.75	1.92	48.20	30.39	16.65	14.76	15.64	13.86	11.41	8.91	7.30	6.95	3.86	3.39	1.72	2.39	10.96	12.32
H3×6	1.87	1.21	1.79	1.26	30.16	21.70	16.69	13.46	15.55	13.25	11.61	8.57	8.58	7.17	3.52	3.11	2.13	2.75	10.13	12.73
H3×7	1.60	1.47	2.89	1.21	42.09	20.67	15.94	15.48	14.76	14.05	10.66	9.94	7.77	6.63	3.51	3.58	1.73	2.43	10.68	12.67
H3×8	1.42	1.11	2.75	2.42	38.48	29.37	14.18	12.78	14.32	12.78	9.46	7.64	6.88	6.16	3.64	3.32	2.02	2.63	11.10	12.06
H3×9	2.51	1.78	1.80	1.43	45.84	29.92	16.96	13.22	17.63	16.15	10.88	7.99	10.13	9.31	3.83	3.33	1.71	2.30	10.25	11.52
H3×10	1.08	.76	3.00	2.12	27.24	16.77	15.30	13.05	13.46	11.47	10.37	8.61	8.33	6.91	2.60	2.53	1.64	2.28	11.56	12.38
H3×11	2.62	1.64	2.03	1.63	53.21	30.82	20.12	15.86	17.25	14.50	14.08	9.70	9.60	7.78	4.09	3.50	1.71	2.25	11.07	11.69
Till-Toroq (P4)	1.97	1.15	2.13	1.27	44.03	17.74	16.40	13.13	16.04	13.02	10.79	8.14	8.49	6.95	3.96	3.07	2.52	3.23	10.98	14.27
H4×5	2.50	1.62	2.38	1.76	59.49	33.92	16.03	14.11	17.79	15.53	11.04	9.11	9.96	8.89	3.93	3.27	1.73	2.26	9.42	10.30
H4×6	1.78	.97	2.75	2.08	47.13	21.89	14.55	11.74	15.53	13.29	10.13	7.50	8.78	7.33	3.75	3.16	1.90	2.47	9.38	11.43
H4×7	1.61	1.29	2.47	1.70	39.75	27.02	14.27	12.63	14.94	13.84	9.52	7.58	8.22	7.65	3.54	2.93	1.93	2.51	9.37	11.64
H4×8	1.75	1.57	2.32	1.92	40.35	29.32	13.59	12.79	15.57	15.00	8.66	7.68	7.88	7.98	3.79	3.57	1.84	2.39	12.32	12.61
H4×9	2.44	1.48	1.86	1.35	43.81	24.01	13.82	10.90	18.83	16.05	8.52	6.38	10.15	8.49	4.32	3.67	1.94	2.41	10.32	11.58
H4×10	1.09	.94	1.80	1.25	19.58	20.58	14.68	14.29	12.86	11.81	10.71	9.98	8.22	7.20	2.47	2.32	1.40	1.91	10.47	11.15
H4×11	2.92	2.39	2.00	1.38	67.17	37.30	17.42	15.79	19.70	19.03	12.15	10.11	11.59	10.35	4.19	3.89	1.56	2.13	11.00	11.32
Savehie (P5)	1.60	1.20	2.65	1.38	40.58	19.00	12.36	10.08	15.92	14.40	8.31	7.17	9.55	8.27	3.34	3.09	2.01	2.70	9.82	11.96
H5×6	2.34	1.87	2.71	1.29	63.19	28.92	15.68	14.76	17.79	16.01	10.62	8.84	10.02	8.86	3.93	3.64	1.90	2.53	9.15	10.35
H5×7	1.31	1.25	2.75	1.60	34.92	21.51	13.00	11.56	15.76	14.11	9.04	7.11	9.32	7.92	3.34	3.19	2.03	2.75	9.72	11.28
H5×8	1.85	1.43	2.29	1.63	40.72	27.29	13.25	11.25	16.69	15.41	8.78	7.00	9.36	8.60	3.65	3.43	2.15	2.46	11.07	11.61
H5×9	2.12	1.52	1.65	1.28	31.89	30.11	13.10	13.31	16.35	15.65	8.33	7.32	9.31	9.18	3.57	3.33	1.73	2.40	8.51	9.53
H5×10	1.12	.96	2.23	1.92	24.05	21.67	13.59	12.41	12.85	12.63	10.11	8.34	8.31	7.75	2.33	2.73	1.51	2.20	11.32	11.88
H5×11	2.13	1.60	2.25	1.67	48.43	30.33	15.22	12.38	17.19	15.98	9.87	7.59	10.64	9.34	4.28	3.42	1.87	2.38	9.33	10.67
Sabouni (P6)	2.28	1.52	1.68	1.31	35.02	22.78	17.77	14.67	16.27	14.44	12.04	9.05	9.08	7.67	3.87	3.51	2.10	3.41	11.59	14.98
H6×7	2.30	1.47	2.52	1.75	40.35	29.42	17.47	13.68	17.39	13.98	11.45	8.41	9.25	7.39	4.02	3.42	1.87	2.40	9.50	11.11
H6×8	2.29	1.48	1.67	1.33	39.25	23.85	16.69	13.00	17.44	14.82	10.66	7.66	9.49	7.94	3.97	3.50	1.95	2.45	10.72	12.02
H6×9	2.28	1.43	2.13	1.38	49.08	22.21	14.58	12.91	17.64	15.02	9.38	7.48	11.10	8.78	3.45	3.25	1.64	2.32	8.39	10.65
H6×10	1.31	.81	2.03	1.60	24.76	14.72	16.45	13.97	13.52	11.84	11.93	9.37	8.57	7.16	3.06	2.48	1.59	2.34	10.50	11.30
H6×11	3.04	1.76	1.83	1.04	56.92	25.78	16.96	14.41	18.34	15.46	10.80	9.13	9.87	8.37	4.23	3.65	1.97	2.37	10.71	11.69
Samsouri (P7)	1.22	.94	2.94	1.82	32.51	20.64	12.99	11.01	14.38	12.60	8.24	6.60	7.64	6.82	3.11	2.81	2.85	3.47	10.96	12.80
H7×8	1.21	.90	2.72	1.97	30.92	20.27	12.38	10.58	14.39	12.72	7.71	6.30	7.30	6.84	3.43	2.82	2.47	3.03	11.82	13.77
H7×9	2.27	1.26	2.20	1.96	47.62	27.29	13.30	11.81	18.27	15.28	8.02	6.73	11.22	9.51	3.68	2.96	1.77	2.29	8.48	9.92
H7×10	0.81	0.59	3.04	2.42	25.79	15.98	12.64	11.40	11.59	10.17	8.96	7.50	7.19	5.95	2.31	2.02	1.56	2.21	10.12	10.60
H7×11	1.90	1.06	1.75	1.28	33.47	15.45	13.76	11.87	16.38	14.41	8.94	7.45	9.27	8.52	3.68	2.92	1.67	2.26	9.27	11.00
Laki (P8)	1.21	.86	2.50	1.93	29.32	19.90	11.77	10.14	14.39	12.84	7.19	6.00	6.95	6.86	3.76	2.83	2.09	2.58	12.68	14.75
H8×9	2.13	1.30	1.79	1.38	38.10	21.28	13.21	10.49	17.35	14.43	7.65	5.47	10.09	8.25	4.08	3.21	2.07	2.73	9.86	11.17
H8×10	.92	1.02	2.58	2.25	23.49	20.78	13.12	12.62	12.17	12.21	9.60	8.46	7.29	6.75	2.50	2.62	1.84	2.40	13.23	13.64
H8×11	1.74	1.18	1.85	1.66	29.93	19.66	12.96	11.51	15.71	14.15	8.04	6.85	7.83	7.93	3.71	3.06	1.94	2.94	10.56	13.25
Dastjerdi (P9)	2.22	1.23	1.89	1.55	44.83	21.22	13.03	9.91	19.10	14.55	7.12	5.32	11.54	9.04	4.04	2.78	2.47	3.09	8.78	10.45
H9×10	1.39	1.02	2.50	1.46	32.93	17.60	12.10	11.18	15.63	13.49	7.79	6.37	10.70	8.47	2.67	2.65	1.52	2.15	10.12	11.70
H9×11	3.07	1.77	1.41	1.05	45.08	26.40	15.93	12.50	20.82	16.30	9.82	7.43	12.53	9.89	4.40	3.20	1.70	2.52	8.87	10.28
Gargar (P10)	0.90	0.64	2.86	1.67	25.70	17.96	12.97	9.93	13.07	11.56	8.05	6.32	8.80	7.93	2.34	2.11	2.25	2.77	10.61	11.95
H10×11	2.07	1.40	1.71	1.20	33.24	21.41	22.44	18.58	15.29	12.67	17.55	14.08	9.19	7.50	2.99	2.53	1.61	2.22	10.99	11.45
Majidi-Ab (P11)	2.86	1.49	1.48	1.15	38.92	19.42	15.60	12.89	19.48	15.47	9.79	7.70	11.45	9.01	4.12	3.29	2.95	3.63	9.83	11.74
LSD (0.05)	0.44	0.4	0.69	0.58	8.83	6.67	1.8	1.68	2.03	1.78	1.51	1.38	1.58	1.08	0.57	0.49	0.43	0.41	1.05	0.9

Supplementary Table 1. Mean comparisons of measured traits in melon parents and hybrids, cultivated in non-saline (N) and saline (S) field conditions. FWT; fruit weight; NF, number of fruit; FY, yield; FL, fruit length; FW, fruit width; SCL, seed cavity length; SCW, seed cavity width; FT, fruit flesh thickness; PT, fruit peel thickness; TSS, fruit total soluble solids.

Genotype	FWT		NF		FY		FL		FW		SCL		SCW		FT		PT		TSS	
	MP	BP	MP	BP	MP	BP	MP	BP	MP	BP	MP	BP	MP	BP	MP	BP	MP	BP	MP	BP
H1×2	0	-23	-2.1	-22.7	0	-7.2	0	-8.6	-4	-11.7	2	8.0	5	18.9	1	-11.1	1	-2.3	-0.9	-8.0
H1×3	10	-17.2	6.5	-9.1	25.8	4.3	1.7	-0.4	2.9	-11.2	5.7	6.8	5.2	31.5	0.9	-12.0	-21.9	-31.8	-2.4	-3.7
H1×4	-6.8	-17.3	3	-6.3	-3.8	-4.9	-3.0	-5.9	-1.3	-8.1	3.8	8.6	-7.1	5.7	2.8	0.1	-14.1	-25.6	-6.3	-8.9
H1×5	15.8	-5.8	-9.1	-24.5	10.5	5	9.1	-1.8	6.1	-1.5	4.9	14.8	3.4	10.4	4.8	-5.8	-2.5	-6.5	-4.2	-11.6
H1×6	12	6.2	10.7	8.6	10.2	-2.2	2.6	-4.1	4.9	-1.7	6.4	18.0	8.0	18.5	-0.5	-4.1	-2.4	-8.2	-0.3	-0.4
H1×7	22.4	-9.6	-14.8	-32.1	11	-4.5	5.9	-2.5	4.1	-7.8	12.6	23.8	8.6	31.4	2.3	-10.7	-15.1	-30.0	-2.2	-5.0
H1×8	-17.2	-39	-29.4	-40	-22.5	-36.1	-1.8	-13.5	-8.7	-19.1	-0.6	18.0	-10.1	15.1	-9.3	-13.9	-6.8	-12.1	-12.8	-16.4
H1×9	3.2	-3.4	23.6	18.9	37.3	36.8	-2.7	-10.3	8.8	7.4	9.8	31.1	17.1	20.9	-7.4	-8.9	-22.1	-31.8	-2.9	-14.8
H1×10	-29.6	-52.4	31.9	6.3	-9.1	-28.7	-1.9	-9.7	-9.7	-23.2	14.7	27.7	-5.3	5.7	-11.9	-31.3	-25.0	-31.6	2.9	-1.6
H1×11	-38.6	-41.9	24	14.3	-13	-19	-4.1	-4.6	-14.0	-15.9	-11.0	-10.6	-13.6	-11.2	-8.8	-9.4	-11.7	-28.2	-11.7	-18.5
H2×3	7.3	4	-40.9	-46.3	-18.4	-27.8	5.2	-2.1	1.0	-1.5	9.0	19.0	-3.9	0.3	-0.8	-2.9	-26.0	-34.0	-0.6	-6.6
H2×4	17	-0.7	9.9	-6.3	29.5	21.5	1.0	-10.2	6.5	0.5	3.0	16.8	-1.9	2.1	13.5	3.4	-7.9	-18.5	-2.6	-7.2
H2×5	27	18.3	-39.5	-43.3	-17.7	-19.7	6.4	4.7	7.8	2.1	5.2	6.4	2.6	13.6	9.9	8.4	7.6	5.6	-2.3	-3.0
H2×6	28.1	2.6	25.3	-2.5	61.8	54.3	4.8	-9.9	11.7	4.7	8.3	30.8	6.4	14.7	-2.6	-10.5	-2.8	-6.5	-7.7	-14.2
H2×7	3.3	-2.7	-28.8	-29.8	-17.4	-23.9	.8	-0.1	2.5	2.0	5.7	7.4	5.1	6.6	0.7	-1.3	-13.7	-27.5	8.1	3.2
H2×8	16	8.9	-14	-21.5	-0.2	-12.2	4.7	0.6	2.4	1.9	14.2	24.7	11.2	18.6	-3.5	-10.0	5.7	1.8	-13.7	-23.0
H2×9	31.7	6.6	7.5	-12.6	49.4	39	12.5	11.4	6.6	-7.0	11.4	22.3	0.5	24.0	8.7	-2.0	-19.1	-27.8	3.6	-2.5
H2×10	44.8	19.6	-21.8	-24	19.1	-0.7	47.1	46.0	4.9	0.6	80.2	85.4	2.0	8.1	-11.8	-24.1	-14.4	-20.3	0.2	-2.9
H2×11	1.8	-24.6	27	-5.5	21.8	21.2	4.9	-4.6	-11.4	-23.3	4.1	11.9	-5.9	15.5	1.2	-9.6	-33.0	-44.5	-7.3	-7.9
H3×4	0.6	-16.8	-9.2	-15.5	-8.5	-23.4	-5.6	-10.2	-4.5	-12.0	-3.4	0	-5.2	3.1	-3.3	-13.7	-11.9	-12.6	0.8	-0.8
H3×5	31.7	19.1	7.3	3.8	37.2	18.8	22.6	12.4	6.2	-1.8	24.2	37.4	-12.9	1.0	19.8	15.6	-23.4	-30.6	3.7	-3.2
H3×6	4.5	-18.2	-13.9	-27.7	-6.8	-13.9	2.5	-6.1	4.4	-4.4	5.0	15.2	5.2	18.7	0.9	-9.1	-6.7	-13.8	-11.6	-12.7
H3×7	27.6	23.9	6.5	-2	35.4	29.5	14.7	7.6	5.8	2.6	16.5	29.5	4.5	7.5	12.8	12.6	-35.0	-39.2	-4.1	-5.6
H3×8	13.4	9.8	10.5	10	30.4	29.6	6.7	-4.2	2.6	-0.5	9.7	31.7	-3.0	-1.1	6.1	-3.1	-11.7	-18.6	-7.5	-12.4
H3×9	42.6	12.7	-17.6	-27.3	23	2.2	21.9	14.5	8.1	-7.7	26.5	52.7	7.9	40.1	7.3	-5.2	-30.9	-31.1	2.0	-9.5
H3×10	-1.1	-16.2	12.4	4.9	-1.7	-8.3	10.2	3.3	1.2	-0.4	14.4	28.8	4.0	15.3	-4.6	-16.3	-30.6	-33.9	5.4	2.1
H3×11	26.3	-8.3	2.9	-17.9	55.1	36.7	32.3	29.0	4.5	-11.5	41.8	43.9	2.8	32.8	13.3	-0.7	-37.0	-42.1	4.7	-2.2
H4×5	40.2	26.9	-0.7	-10.4	40.6	35.1	11.5	-2.3	11.3	10.9	15.6	32.9	10.5	17.3	7.8	-0.6	-23.7	-31.4	-9.4	-14.1
H4×6	-16.3	-22	44.1	28.9	19.2	7	-14.8	-18.1	-3.9	-4.6	-11.3	-6.2	-0.1	3.3	-4.1	-5.1	-17.6	-24.5	-16.9	-19.1
H4×7	1	-18.4	-2.7	-16.1	3.9	-9.7	-2.8	-12.9	-1.8	-6.9	0	15.5	1.9	7.6	0.2	-10.4	-28.1	-32.3	-14.5	-14.6
H4×8	10.1	-11.2	0.2	-7.1	10	-8.4	-3.5	-17.1	2.3	-2.9	-3.7	20.4	2.0	13.3	-1.7	-4.2	-20.2	-27.0	4.1	-2.9
H4×9	16.4	9.8	-7.7	-12.9	-1.4	-2.3	-6.1	-15.7	7.2	-1.4	-4.8	19.7	1.4	19.6	7.9	6.8	-22.0	-22.8	4.5	-6.0
H4×10	-24.1	-44.8	-27.9	-37.1	-43.8	-55.5	.0	-10.5	-11.6	-19.8	13.7	33.0	-4.9	-3.2	-21.5	-37.5	-41.3	-44.5	-3.0	-4.6
H4×11	20.8	2.1	10.9	-6.3	62	52.6	8.9	6.2	10.9	1.1	18.1	24.2	16.2	36.5	3.6	1.5	-42.9	-47.1	5.8	0.3
H5×6	20.4	2.3	25	2.2	67.2	55.7	4.1	-11.8	10.5	9.3	4.4	27.8	7.6	10.4	9.1	1.6	-7.5	-9.5	-14.5	-21.1
H5×7	-7.2	-18.3	-1.7	-6.6	-4.5	-13.9	2.6	0.1	4.0	-1.0	9.3	9.8	8.4	21.9	3.6	0.2	-16.4	-28.7	-6.4	-11.3
H5×8	31.9	15.9	-11	-13.5	16.5	0.4	9.9	7.2	10.1	4.8	13.3	22.2	13.5	34.7	2.8	-2.9	4.7	2.8	-1.6	-12.7
H5×9	10.7	-4.9	-27.3	-37.7	-25.3	-28.9	3.2	0.5	-6.7	-14.4	7.9	16.9	-11.7	-2.5	-3.3	-11.8	-22.7	-29.8	-8.5	-13.4
H5×10	-10.2	-29.9	-19.1	-22.1	-27.4	-40.7	7.3	4.8	-11.4	-19.3	23.5	25.5	-9.5	-5.6	-17.8	-30.0	-28.9	-32.5	10.9	6.7
H5×11	-4.4	-25.5	9.1	-15.1	21.8	19.4	8.9	-2.4	-2.9	-11.8	9.1	18.8	1.4	11.5	14.7	3.7	-24.9	-36.8	-5.1	-5.1
H6×7	31.3	0.6	8.8	-14.5	19.5	15.2	13.6	-1.7	13.4	6.8	12.9	39.0	10.6	21.0	15.0	3.8	-24.3	-34.2	-15.8	-18.1
H6×8	30.8	0	-20.3	-33.3	22	12.1	13.0	-6.1	13.7	7.1	10.8	48.3	18.4	36.5	4.0	2.5	-6.9	-7.1	-11.7	-15.5
H6×9	1.1	-0.3	19.4	12.8	22.9	9.5	-5.3	-18.0	-.3	-7.6	-2.1	31.8	7.7	22.2	-12.8	-14.7	-28.3	-33.7	-17.7	-27.6
H6×10	-17.7	-42.7	-10.9	-29.2	-18.5	-29.3	7.0	-7.5	-7.8	-16.9	18.7	48.1	-4.2	-2.7	-1.5	-20.9	-26.8	-29.1	-5.4	-9.5
H6×11	18.1	6.2	16.1	9	54	46.2	1.7	-4.6	2.6	-5.9	-1.1	10.3	-3.9	8.6	5.8	2.5	-22.1	-33.3	0	-7.6
H7×8	0	-0.3	0	-7.5	0	-4.9	.0	-4.7	0	0	0	7.3	0	5.0	0	-8.5	0	-13.4	0	-6.8
H7×9	31.9	2	-9	-25.3	23.1	6.2	2.3	2.1	9.2	-4.3	4.4	12.6	16.9	46.7	2.7	-9.1	-33.5	-37.9	-14.1	-22.7
H7×10	-23.8	-33.8	4.7	3.2	-11.4	-20.7	-2.6	-2.7	-15.6	-19.4	10.0	11.3	-12.6	-5.9	-15.5	-26.0	-38.9	-45.4	-6.1	-7.6
H7×11	-6.6	-33.4	-20.8	-40.6	-6.3	-14	-3.7	-11.8	-3.3	-15.9	-0.8	8.5	-2.9	21.3	1.7	-10.8	-42.4	-43.4	-10.8	-15.4
H8×9	24.2	-4.1	-18.4	-28.3	2.8	-15	6.5	1.4	3.6	-9.1	7.0	7.5	9.2	45.2	4.8	1.1	-9.4	-16.3	-8.1	-22.3
H8×10	-12.8	-24	-3.9	-10	-14.6	-19.9	6.1	1.1	-11.4	-15.4	26.0	33.5	-7.4	5.0	-18.1	-33.5	-14.9	-17.8	13.6	4.3
H8×11	-14.3	-39	-6.9	-26	-12.3	-23.1	-5.3	-16.9	-7.3	-19.4	-5.2	11.9	-14.8	12.7	-5.9	-10.0	-23.2	-34.5	-6.2	-16.7
H9×10	-10.7	-37.3	5.2	-12.6	-6.6	-26.5	-6.9	-7.1	-2.8	-18.1	2.7	9.4	5.2	21.6	-16.3	-33.9	-35.6	-38.5	4.4	-4.6
H9×11	20.9	7.4	-16.2	-25.4	7.7	0.6	11.3	2.2	7.9	6.9	16.1	37.9	9.0	9.4	7.8	6.8	-37.2	-42.4	-4.7	-9.8
H10×11	10.1	-27.7	-21	-40.1	2.9	-14.6	57.1	43.8	-6.0	-21.5	96.8	118.0	-9.3	4.3	-7.5	-27.4	-37.9	-45.3	7.5	3.6
Mean	8.5	-9.3	-2.1	-14	10.2	-0.01	5.9	-1.5	1.2	-6.1	11.2	23.7	1.5	14.2	0.1	-8	-19.7	-26.2	-3.6	-8.9
LSD (0.05)	0.4	0.4	0.6	0.7	7.7	8.8	1.6	1.8	1.8	2	1.3	1.5	1.4	1.6	0.5	0.6	0.4	0.4	0.9	1.1
LSD (0.01)	0.5	0.6	0.8	0.9	10.1	11.7	2.1	2.4	2.3	2.7	1.7	2	1.8	2.1	0.7	0.8	0.5	0.6	1.2	1.4

Supplementary Table 2. Heterosis (%) values based on mid-parent (MP) and best parent (BP) for measured traits in non-saline conditions. FWT; fruit weight; NF, number of fruit; FY, yield; FL, fruit length; FW, fruit width; SCL, seed cavity length; SCW, seed cavity width; FT, fruit flesh thickness; PT, fruit peel thickness; TSS, fruit total soluble solids.

Genotype	FWT		NF		FY		FL		FW		SCL		SCW		FT		PT		TSS	
	MP	BP	MP	BP	MP	BP	MP	BP	MP	BP	MP	BP	MP	BP	MP	BP	MP	BP	MP	BP
H1×2	0	-24.2	-3.3	-29.4	0	-4.1	0	-9.1	0.9	-13.2	1	9.6	1.1	27.1	2.1	-8.9	-2.0	-7.1	0.9	-2.8
H1×3	40.8	-0.5	-37.4	-49.7	6.1	-7.9	26.4	22.8	16.3	-4.3	40.5	42.9	15.7	57.8	18.4	0.9	-14.8	-21.7	-9.2	-17.3
H1×4	5.3	-16.5	10.8	7.9	9.0	-8.4	4.5	4.3	7.9	-5.5	9.9	13.5	11.9	41.5	3.6	-3.3	-11.5	-21.2	-14.5	-21.0
H1×5	-17.4	-33.5	16.8	9.7	13.8	-1.7	5.5	-6.6	-3.4	-11.5	10.9	14.5	-2.5	11.4	13.3	6.3	-5.3	-8.4	-7.0	-7.6
H1×6	-33.5	-40.9	16.3	11.6	-12.6	-18.1	-13.9	-18.6	-9.3	-16.9	-13.5	-5.6	-7.9	9.9	-16.1	-16.3	-17.2	-28.0	2.3	-7.5
H1×7	5.1	-22.4	5.7	-12.0	-11.6	-20.8	4.9	-3.4	0.2	-13.4	10.9	19.5	-1.4	26.1	2.6	-7.9	-16.7	-28.1	2.9	0
H1×8	-11.7	-36.4	-15.4	-31.2	-16.8	-26.7	1.7	-9.7	-3.6	-16.1	-6.8	5.9	-7.3	18.1	0.2	-9.8	-8.7	-9.7	4.6	-4.8
H1×9	3.2	-15.9	17.8	4.8	19.3	8.2	11.6	-1.9	3.1	-5.2	22.6	49.3	0.1	8.8	6.9	-4.4	-18.0	-25.6	-4.6	-11.1
H1×10	-45.1	-63.6	28.7	11.0	-31.2	-41.9	-2.7	-14.4	-15.7	-29.7	2.4	13.1	-13.4	1.3	-24.2	-39.5	-25.7	-29.0	-3.2	-3.8
H1×11	-12.9	-23.4	-15.1	-17.2	-25.8	-35.3	-5.6	-6.2	-4.8	-9.9	-0.7	-0.3	2.1	11.2	-17.2	-20.0	-12.6	-25.9	-1.7	-3.2
H2×3	-14.6	-23.1	-41.8	-48.9	-13.7	-27.7	4.3	-2.5	-1.6	-7.6	3.0	15.0	-2.3	3.6	-2.8	-9.7	-13.4	-16.2	-11.8	-21.6
H2×4	16.6	9.7	-29.5	-47.7	-16.4	-32.1	-3.9	-12.8	10.5	9.3	-5.9	6.8	7.5	7.9	13.3	10.2	-10.9	-16.7	-2.8	-12.5
H2×5	8.7	0.3	-32.8	-48.8	-16.5	-30.3	8.4	5.3	3.8	-2.2	7.5	13.8	5.7	16.3	4.5	1.3	1.2	-0.9	-7.4	-9.4
H2×6	13.5	-5.6	-36.6	-52.4	-25.0	-32.4	4.4	-9.7	4.3	-1.8	10.1	32.8	7.4	13.5	-1.8	-10.3	-19.9	-26.8	-12.2	-22.6
H2×7	0	-3.8	-19.0	-31.5	0	-13.7	.0	-1.4	0	-0.6	1	1.5	0	1	-1	-1.7	0	-9.5	-1	-5.3
H2×8	38.0	27.8	-42.2	-49.9	-12.2	-25.3	13.6	10.6	11.7	11.2	11.9	15.7	11.2	11.4	20.2	18.6	-0.6	-4.8	-1.1	-12.2
H2×9	74.0	58.4	-4.2	-23.9	78.7	56.1	31.1	26.3	22.9	15.2	28.3	41.4	19.9	38.5	29.5	26.9	-17.1	-20.8	3.4	-1.0
H2×10	59.9	30.3	-38.7	-49.9	-10.0	-26.5	67.5	61.5	6.3	1.4	101.8	103.4	8.0	16.1	1.9	-12.0	-16.5	-17.2	-8.1	-10.1
H2×11	41.5	18.8	-7.3	-33.4	31.2	10.5	13.9	4.2	11.2	1.4	16.8	28.5	13.8	31.3	11.1	4.5	-25.2	-33.6	-5.5	-6.7
H3×4	0	-14.7	6.1	-12.9	0	-3.7	0	-3.1	-3	-7.0	0.5	1.5	1	6.5	-4	-9.4	-1	-3.5	0.8	-0.7
H3×5	83.3	53.8	14.0	-3.6	59.3	58.6	31.8	19.9	8.3	-3.8	18.3	24.4	-3.6	13.0	21.5	9.7	-16.4	-20.7	-7.6	-16.2
H3×6	3.8	-20.5	-24.0	-36.8	3.5	-4.8	-0.2	-8.2	3.4	-8.2	1.1	8.4	3.7	16.5	3.8	-11.3	-14.4	-19.4	-14.2	-15.0
H3×7	68.6	57.5	-36.5	-39.2	3.9	0.2	32.7	25.7	18.1	11.5	37.0	50.5	2.1	7.7	35.1	27.5	-25.0	-30.0	-7.9	-13.9
H3×8	32.1	28.2	23.5	21.6	50.4	47.6	13.9	3.8	6.3	-0.5	9.9	27.4	-5.3	0.1	25.1	17.6	-6.0	-12.7	-18.1	-18.3
H3×9	74.1	44.4	-19.4	-28.3	48.2	41.0	19.0	7.4	25.4	11.0	20.8	50.0	22.6	51.4	26.5	19.8	-24.7	-25.7	-8.4	-21.6
H3×10	5.1	-6.2	15.8	6.5	-9.6	-12.5	17.3	6.0	0.8	-0.8	21.1	36.3	-1.9	12.3	9.9	1.5	-21.2	-24.4	-7.1	-15.9
H3×11	42.7	10.2	3.6	-18.2	59.8	58.7	25.9	23.1	8.8	-6.3	24.4	26.0	2.6	26.4	21.1	6.3	-32.1	-37.9	-11.6	-20.5
H4×5	38.3	35.5	33.2	28.3	84.6	78.5	21.6	7.5	13.3	7.9	19.0	27.1	16.8	27.9	6.2	5.8	-23.7	-30.0	-21.5	-27.8
H4×6	-27.5	-36.5	61.0	58.5	8.0	-3.9	-15.5	-20.0	-3.2	-7.9	-12.7	-7.8	0.3	5.6	-4.0	-10.1	-25.7	-27.6	-21.9	-23.7
H4×7	23.3	11.9	9.9	-6.5	40.8	31.0	4.6	-3.8	8.1	6.3	2.8	14.7	11.0	12.1	-0.2	-4.5	-25.0	-27.6	-14.0	-18.4
H4×8	56.2	36.8	19.8	-0.4	55.8	47.3	9.9	-2.6	16.0	15.2	8.6	28.0	15.6	16.4	21.1	16.3	-17.7	-26.1	-13.1	-14.5
H4×9	24.1	19.8	-4.4	-12.9	23.2	13.2	-5.4	-17.0	16.4	10.3	-5.3	19.8	6.2	22.2	25.4	19.6	-23.7	-25.4	-6.3	-18.9
H4×10	4.7	-18.6	-15.0	-25.0	15.3	14.6	23.9	8.8	-3.9	-9.3	38.0	57.9	-3.2	3.6	-10.5	-24.5	-36.5	-41.1	-14.9	-21.9
H4×11	81.2	60.5	13.5	7.9	100	92.0	21.3	20.2	33.6	23.0	27.6	31.2	29.7	49.0	22.4	18.2	-38.0	-41.3	-13.0	-20.7
H5×6	37.4	22.7	-4.0	-6.1	38.4	26.9	19.3	0.6	11.1	10.9	9.0	23.3	11.2	15.5	10.2	3.7	-17.2	-25.9	-23.2	-30.9
H5×7	17.2	4.4	0.1	-12.1	8.5	4.2	9.6	4.9	4.6	-2.0	3.3	7.7	5.0	16.1	8.2	3.2	-10.8	-20.7	-8.9	-11.9
H5×8	39.1	19.6	-1.5	-15.6	40.3	37.1	11.3	11.0	13.2	7.0	6.3	16.7	13.6	25.3	15.8	10.8	-6.9	-9.0	-13.0	-21.3
H5×9	25.2	23.4	-12.3	-17.2	49.8	41.9	33.1	32.0	8.1	7.6	17.2	37.5	6.0	11.0	13.3	7.7	-17.0	-22.2	-15.0	-20.4
H5×10	4.6	-19.9	26.0	15.0	17.3	14.1	24.0	23.0	-2.7	-12.3	23.7	32.0	-4.3	-2.3	5.1	-11.6	-19.4	-20.3	-0.6	-0.7
H5×11	18.9	7.3	32.1	21.2	57.9	56.1	7.7	-4.0	7.0	3.3	2.1	6.0	8.1	13.0	7.2	4.0	-24.9	-34.5	-10.0	-10.8
H6×7	19.7	-3.4	11.7	-3.8	35.5	29.1	6.5	-6.7	3.5	-3.1	7.4	27.3	1.9	8.3	8.2	-2.7	-30.4	-31.0	-20.0	-25.8
H6×8	24.4	-2.6	-17.7	-30.7	11.7	4.7	4.8	-11.4	8.7	2.7	1.8	27.7	9.2	15.7	10.6	-0.2	-18.1	-28.1	-19.1	-19.8
H6×9	3.6	-6.3	-4.0	-11.3	0.9	-2.5	5.0	-12.0	3.6	3.2	4.0	40.5	5.1	14.5	3.3	-7.4	-28.8	-32.1	-16.3	-28.9
H6×10	-24.8	-46.6	7.3	-4.0	-27.7	-35.4	13.6	-4.8	-8.9	-18.0	22.0	48.4	-8.3	-6.7	-11.7	-29.3	-24.1	-31.3	-16.0	-24.5
H6×11	16.5	15.1	-15.4	-20.8	22.2	13.2	4.5	-1.8	3.4	0	9.0	18.5	0.4	9.1	7.4	4.1	-32.7	-34.7	-12.5	-21.9
H7×8	0	-3.9	5.3	2.4	0	-1.8	1	-4.0	2	-0.9	0	5.0	2	0.3	1	-0.3	-6	-12.9	-3	-6.6
H7×9	15.8	1.8	16.3	7.7	30.4	28.6	12.8	7.2	12.5	5.0	12.8	26.3	19.9	39.4	6.1	5.6	-30.2	-34.1	-14.7	-22.5
H7×10	-24.6	-36.6	38.7	32.9	-17.2	-22.6	8.8	3.5	-15.8	-19.3	16.0	18.7	-19.4	-12.8	-17.9	-28.1	-29.2	-36.4	-14.3	-17.2
H7×11	-12.8	-29.0	-14.1	-29.9	-22.8	-25.1	-0.7	-8.0	2.7	-6.8	4.1	12.8	7.6	24.9	-4.2	-11.3	-36.4	-37.7	-10.4	-14.1
H8×9	23.9	5.2	-20.4	-28.1	3.5	0.3	4.7	3.5	5.4	-0.8	-3.4	2.7	3.8	20.2	14.5	13.6	-3.7	-11.6	-11.4	-24.3
H8×10	35.8	18.1	25.3	16.9	9.8	4.4	25.8	24.5	0.1	-4.9	37.4	41.0	-8.7	-1.6	6.1	-7.4	-10.1	-13.2	2.2	-7.5
H8×11	0	-21.0	8.1	-13.7	0	-1.2	1	-10.7	0	-8.5	4	14.2	3	15.6	-1	-7.1	-5.4	-19.0	-4	-10.2
H9×10	8.6	-17.6	-9.3	-12.5	-10.2	-17.1	12.7	12.6	3.4	-7.3	9.4	19.6	-0.2	6.7	8.4	-4.7	-26.6	-30.4	4.5	-2.1
H9×11	29.6	18.5	-21.9	-32.1	29.9	24.4	9.6	-3.0	8.6	5.4	14.0	39.5	9.6	9.8	5.2	-2.9	-25.1	-30.6	-7.4	-12.4
H10×11	31.3	-6.2	-14.7	-28.0	14.5	10.2	62.9	44.1	-6.3	-18.1	100	123	-11.5	-5.5	-6.3	-23.2	-30.5	-38.8	-3.3	-4.2
Mean	17.6	0.7	-1.6	-12.9	14.6	6.7	11.8	3.8	5	-2.1	13.8	25.5	3.9	15.3	6.5	-1.4	-18	-23.6	-8.4	-14.3
LSD (0.05)	0.3	0.4	0.5	0.6	5.8	6.7	1.5	1.7	1.5	1.8	1.2	1.4	0.9	1.1	0.4	0.5	0.4	0.4	0.8	0.9
LSD (0.01)	0.5	0.5	0.7	0.8	7.7	8.9	1.9	2.2	2	2.4	1.6	1.8	1.2	1.4	0.6	0.7	0.5	0.5	1	1.2

Supplementary Table 3. Heterosis (%) values based on mid-parent (MP) and best parent (BP) for measured traits of hybrids in saline conditions. FWT; fruit weight; NF, number of fruit; FY, yield; FL, fruit length; FW, fruit width; SCL, seed cavity length; SCW, seed cavity width; FT, fruit flesh thickness; PT, fruit peel thickness; TSS, fruit total soluble solids.
